# Comparative efficacy and safety of all kinds of intraocular lenses in presbyopia-correcting cataract surgery: a systematic review and meta-analysis

**DOI:** 10.1186/s12886-024-03446-1

**Published:** 2024-04-16

**Authors:** Jinyu Li, Bin Sun, Yuexin Zhang, Yansong Hao, Ze Wang, Chengjiang Liu, Shanhao Jiang

**Affiliations:** 1 Department of Ophthalmology, Yantai Affiliated Hospital of Binzhou Medical University, Binzhou Medical University, Jinbu Street, Muping area, Yantai, Shandong Province 264000 China; 2 Department of Ophthalmology, Shijiazhuang People’s Hospital, Shi Jiazhuang, 050200 China; 3 Department of General Medicine, Affiliated Anqing First People’s Hospital of Anhui Medical University, Anhui, 246000 China

**Keywords:** Presbyopia, Cataract, Intraocular lenses, Visual quality

## Abstract

**Purpose:**

To assess the efficacy and safety of various intraocular lenses (IOLs), including standard monofocal, bifocal, trifocal, extended depth of focus (EDOF), and enhanced monofocal IOLs, post-cataract surgery through a network meta-analysis.

**Methods:**

A systematic search of PubMed, Cochrane Library, and Web of Science was conducted to identify relevant studies from the past 5 years. Parameters such as binocular visual acuities, spectacle independence, contrast sensitivity (CS), and optical quality were used to evaluate efficacy and safety. Data from the selected studies were analyzed using Review Manager 5.4 and STATA 17.0 software.

**Results:**

Twenty-eight Randomized Controlled Trials (RCTs) comprising 2465 subjects were included. Trifocal IOLs exhibited superior uncorrected near visual acuity (UNVA) compared to monofocal IOLs (MD: -0.35; 95% CI: -0.48, -0.22). Both trifocal (AcrySof IQ PanOptix IOLs group MD: -0.13; 95% CI: -0.21, -0.06) and EDOF IOLs (MD: -0.13; 95% CI: -0.17, -0.09) showed better uncorrected intermediate visual acuity (UIVA) than monofocal IOLs. Trifocal IOLs ranked highest in spectacle independence at various distances (AT LISAtri 839MP group: SUCRA 97.5% for distance, 80.7% for intermediate; AcrySof IQ PanOptix group: SUCRA 83.0% for near).

**Conclusions:**

For cataract patients who want to treat presbyopia, trifocal IOLs demonstrated better visual acuity and spectacle independence at near distances. Different types of trifocal IOL characteristics differ. EDOF and enhanced monofocal IOLs have improved visual quality at intermediate distances.Therefore, It is very important to select the appropriate IOLs based on the lens characteristics and patient needs.

**Supplementary Information:**

The online version contains supplementary material available at 10.1186/s12886-024-03446-1.

## Introduction

Cataract, characterized by lens opacification, is a leading global cause of blindness and vision impairment [1, 2]. Particularly prevalent in developing countries, it contributes to vision loss in 33% of visually impaired individuals [3]. Approximately 100 million people with moderate-to-severe distance vision impairment or blindness could benefit from cataract surgery [4]. The current standard of care for significant cataracts is phacoemulsification combined with the intraocular lens implantation [5]. However, the increasing variety of lenses pose a significant challenge to ophthalmologists and cataract patients in the clinical choosing. Therefore, it is crucial to assess the function of common IOLs in the clinic.

In the era of increased reliance on electronic devices by middle-aged and elderly individuals, the demand for high-quality vision at intermediate and near distances has surged [6]. As a result, the utilization of high-quality IOLs to address pseudo presbyopia has emerged as a significant subject in contemporary cataract surgery practices [7]. Beyond standard monofocal IOLs, current clinical practice often involves multifocal IOLs (bifocal and trifocal), EDOF IOLs, and enhanced monofocal IOLs [8–10]. While standard monofocal IOLs focus light at a single predetermined distance, necessitating additional refractive correction for varying distances [11]. In contrast, the mechanism of multifocal IOLs achieve a zooming effect through the use of rings with varying refractive capabilities in different areas of the lens, they can offer superior visual acuity at both near and intermediate distances, along with reduced reliance on corrective eyewear [12–15]. In the earlier stages, the first generation of multifocal IOLs were bifocal, featuring only two optical zones and the subsequent emergence of trifocal IOLs containing three optical zones [16, 17]. However, they may compromise optical quality, leading to decreased contrast sensitivity, glare, and halo [18, 19]. EDOF IOLs, endorsed by the American Academy of Ophthalmology, extend the focus area to cover intermediate distances, providing excellent vision for both intermediate and distant ranges. They aim to mitigate the drawbacks of multifocal IOLs, minimizing glare and halos, while cautioning against excessive aberrations that could impact retinal image quality [20, 21]. Moreover, enhanced monofocal IOLs improves intermediate distance vision by a continuous rise in power from the periphery to the center [22]. Therefore it gave better intermediate visual acuity and higher intermediate spectacle independence without impairment of far vision and visual quality, compared to the monofocal IOLs [23]. Although enhanced monofocal IOLs and EDOF IOLs share similarities [24], there is currently a lack of comprehensive comparisons among trifocal, enhanced monofocal, and EDOF IOLs.

Existing studies often lack a thorough comparison of commonly used high-quality IOLs. While one report conducted a network analysis of multiple IOLs, it omitted enhanced monofocal IOLs, limiting the comprehensive assessment of visual quality at mid-distance compared to our study [25]. Our network meta-analysis specifically targets visual quality at intermediate and near distances, providing a comprehensive analysis of various high-quality IOLs. This approach aims to establish a reliable foundation for preoperative patient selection, enhancing postoperative quality of life and satisfaction. The findings of this analysis are expected to encourage the broader adoption of high-quality IOLs in cataract surgery.

## Method

This meta-analysis was guided by the Preferred Reporting Items for Systematic Reviews and Meta-Analyses (PRISMA), which was registered on Prospero with registration code CRD42023456455 [26].

### Search strategy

We searched randomized clinical trials (RCTs) on Cochrane Central Register of Controlled Trials (CENTRAL), PubMed and Embase from 2018 to March 15, 2023. Keywords and Mesh words searched include “Multifocal Intraocular Lenses”, “Lens Implantation, Intraocular”, “Cataract”, “Cataract Extraction”, “Phacoemulsification”.

### Inclusion criteria

Articles meeting the following criteria will be included in conducting the study: (1) Study subjects. Participants underwent cataract surgery in both eyes and had the same IOL implanted in both eyes, and all participants were older than 40 years of age. (2) Intervention measures. Patients in the control group were implanted with standard monofocal IOLs in both eyes. Patients in the treatment group had other types of IOLs implanted in both eyes, including: bifocal IOLs, trifocal IOLs (AT LISAtri 839MP, FineVision POD F, AcrySof IQ PanOptix and other new trifocal IOLs), EDOF IOLs, and enhanced monofocal IOLs. (3) Outcome indicator. All literature covered should report on one or more primary or secondary indicators. The primary outcome indicator were visual acuity, spectacle independence and optical quality. The secondary outcome indicators were as follows: contrast sensitivity (CS) (under photopic and mesopic conditions). (4) Study type. The included studies were RCTs.

### Exclusion criteria

Articles that met the following criteria were excluded: (1) duplicated studies, conference abstracts, letters, review, animal studies or research in vitro. (2) single-arm design studies. (3) cataract surgery in only one eye. (4) different types of IOLs implanted in both eyes. (5) patients had severe irregular astigmatism. (6) patients had ocular pathology that could affect the visual function, history of ocular trauma or prior ocular surgery and IOL centering, intraoperative or postoperative complications and systemic disease.

### Data extraction and quality assessment

Among the included studies, two researchers (J.Y.L. and Y.X.Z.) independently extracted the data. Any discrepancies will be resolved by consensus and, if harmonization is not possible, a third examiner will be consulted for arbitration. We accessed the quality of the included randomized controlled studies using Review Manager 5.4.1 according to the Cochrane risk-of-bias assessment tool. The preoperative data collected included name of the first author [27–54], publication year, sample size, sex, age, follow-up time, interventions. Essential data for the included studies have been extracted and presented in the Table 1.

**Table 1:** The characteristic of included studies

Number	Study	N	Gender(female%)	Age (Mean + SD)	Treatment	Intraocular lens model	Optical Feature	Groups	Follow-up period	Outcomes included in metaanalyses
1	Alió, J. L. (2018) [27]	17	NR	63.2 ± 7.7	bifocal IOL	IOL AT LISA 809 M	diffractive IOL	B	1 m, 6 m	UDVA, CDVA, UNVA, CNVA, UIVA, CIVA, CS under photopic condition, CS under mesopic condition, halo
		17	NR		bifocal IOL	IOL ReSTOR SN6AD1 (Alcon)	diffractive IOL	B		
		15	NR		trifocal-AT LISAtri 839MP	AT LISAtri 839MP	diffractive IOL	C		
2	Cardona, G. (2018) [28]	18	44.40%	72.5 ± 3.5	monofocal IOL	Tecnis ZA9003	aspheric IOL	A	3 m	CIVA
		12	16.67%	77 ± 4.2	bifocal IOL	Tecnis ZKB00	diffractive IOL	B		
		18	38.89%	71.5 ± 3.4	bifocal IOL	Tecnis ZLB00	diffractive IOL	B		
		16	37.50%	72 ± 5.3	bifocal IOL	AcrySof ReSTOR SV25T0	diffractive IOL	B		
		9	100%	69 ± 3.6	bifocal IOL	AT LISA 809 M	diffractive IOL	B		
		6	66.67%	78 ± 4.6	trifocal-AT LISAtri 839MP	AT LISAtri 839MP	diffractive IOL	C		
3	Hogarty, D. T. (2018) [29]	45	51.11%	78.44 ± 8.50	monofocal IOL	aspheric ZA9002 Tecnis® 3-piece IOL or the Tecnis® ZCT IOL	aspheric IOL	A	5 m	UDVA, CDVA, UNVA, CNVA, UIVA, CIVA, SE, Spectacle Independence
		43	55.81%	73.00 ± 6.29	EDOF IOL	Tecnis® Symfony IOL	refractive and diffractive IOL	G		
4	Poyales, F. (2019) [30]	33	66.67%	63.0 ± 7.9	trifocal-FineVision POD F	FineVision POD F	diffractive IOL	D	3 m	UDVA, CDVA, UNVA, CNVA, CIVA, SE, CS under photopic condition, CS under mesopic condition
		30	66.67%	62.0 ± 12.8	trifocal-other new trifocal IOLs	FineVision POD FT	diffractive IOL	F		
5	Lapid-Gortzak, R. (2020) [31]	86	60.67%	66.1 ± 9.3	trifocal-AcrySof IQ PanOptix TFNT00	AcrySof IQ PanOptix TFNT00	diffractive IOL	E	6 m	UDVA, UNVA, UIVA, SE, CS under photopic condition, CS under mesopic condition, halo
		81	61.29%	65.6 ± 9.6	trifocal-AT LISAtri 839MP	AT LISAtri 839MP	diffractive IOL	C		
6	Law, E. M. (2020) [32]	47	NR	NR	monofocal IOL	Bi-Flex 677AB	aspheric IOL	A	3–6 m, 13–18 m	UDVA, CDVA, UNVA, CNVA, CIVA, SE, CS under photopic condition, CS under mesopic condition, halo
		43	NR	NR	bifocal IOL	Bi-Flex 677MY	diffractive IOL	B		
7	Poyales, F. (2020) [33]	24	84.00%	65.0 ± 6.3	trifocal-FineVision POD F	FineVision POD F	diffractive IOL	D	3 m	UDVA, UNVA, CNVA, CIVA, SE, CS under photopic condition, CS under mesopic condition,
		25	88.46%	66.0 ± 6.9	trifocal-other new trifocal IOLs	FineVision POD F GF	diffractive IOL	F		
8	Ribeiro, F. (2020) [34]	15	NR	68 ± 8	trifocal-FineVision POD F	FineVision POD F	diffractive IOL	D	3 m	UDVA, CDVA, UNVA, CNVA, UIVA, CIVA, SE, CS under photopic condition, CS under mesopic condition
		15	NR	66 ± 6	trifocal-other new trifocal IOLs	RayOne Trifocal	diffractive IOL	F		
		15	NR	64 ± 6	trifocal-AcrySof IQ PanOptix TFNT00	AcrySof IQ PanOptix TFNT00	diffractive IOL	E		
9	Ribeiro, F. J. (2020) [35]	30	53.33%	70 ± 8	trifocal-AcrySof IQ PanOptix TFNT00	AcrySof IQ PanOptix TFNT00	diffractive IOL	E	3 m	UDVA, CDVA, UNVA, CNVA, UIVA, CIVA, SE, CS under photopic condition, CS under mesopic condition
		30	60.00%	68 ± 9	trifocal-other new trifocal IOLs	FineVision POD FT	diffractive IOL	F		
10	Webers, V. S. C. (2020) [36]	13	60.00%	70.38 ± 6.0	trifocal-AT LISAtri 839MP	AT LISA tri 839MP	diffractive IOL	C	3 m	UDVA, UNVA, UIVA, SE, CS under photopic condition, CS under mesopic condition, halo, glare
		14	73.33%	67.57 ± 12.21	EDOF IOL	Symfony extended depth-of-focus intraocular lens	refractive and diffractive IOL	G		
11	Auffarth, G. U. (2021) [37]	74	59.46%	72.0 ± 6.8	monofocal IOL	TECNIS Monofocal, Model ZCB00	aspheric monofocal IOL	A	6 m	UDVA, CDVA, UIVA, CIVA, SE, CS under photopic condition, CS under mesopic condition, halo, glare
		68	58.82%	69.3 ± 8.7	enhanced monofocal IOL	The TECNIS Eyhance IOL (Model ICB00)	refractive IOL	H		
12	Messias, A. (2021) [38]	17	NR	NR	monofocal IOL	monofocal SN60WF	aspheric IOL	A	12 m	SE
		16	NR	NR	bifocal IOL	multifocal SN6AD1	diffractive IOL	B		
13	Nováček, L. V. (2021) [39]	12	33.33%	56.0 ± 6.8	trifocal-other new trifocal IOLs	Liberty®677MY	diffractive IOL	F	3 m,12 m	SE, CS under photopic condition, CS under mesopic condition, halo, glare
		16	33.33%	51.0 ± 7.28	trifocal-AT LISAtri 839MP	AT LISA®tri 839 m	diffractive IOL	C		
14	Reinhard, T. (2021) [40]	76	51.30%	68.9 ± 7.63	EDOF IOL	AT LARA 829MP (EDOF)	diffractive IOL	G	6 m	UDVA, CDVA, UIVA, CIVA, UNVA, CNVA, SE, CS under photopic condition, CS under mesopic condition, halo, glare, Spectacle Independence
		66	62.30%	69.4 ± 7.22	EDOF IOL	TECNIS Symfony (EDOF)	refractive and diffractive IOL	G		
		65	63.20%	71.0 ± 6.52	monofocal IOL	CT ASPHINA 409MP	aspheric IOL	A		
15	Tran, D. B. (2021) [41]	25	48.00%	67 ± 8	trifocal-AcrySof IQ PanOptix TFNT00	AcrySof IQ PanOptix TFNT00	diffractive IOL	E	3 m	UDVA, CDVA, UIVA, CIVA, UNVA, CNVA, SE, CS under photopic condition, CS under mesopic condition, halo, glare, Spectacle Independence
		23	73.91%	71 ± 9	EDOF IOL	extended depth of focus (EDOF) IOL	refractive and diffractive IOL	G		
16	Turhan, S. A. (2021) [42]	15	46.67%	63.9 ± 8.4	bifocal IOL	Tecnis + 2.75 D (ZKB00) IOL	diffractive IOL	B	6 m	UDVA, CDVA, UIVA, CIVA, UNVA, CNVA
		14	42.86%	63.4 ± 7.1	EDOF IOL	EDOF Tecnis Symfony (ZXR00) IOL	refractive and diffractive IOL	G		
17	Vargas, V. (2021) [43]	16	NR	60.5 ± 7.0	bifocal IOL	bifocal AcrySof IQ ReSTORþ3.0D	diffractive IOL	B	6 m	UDVA, UIVA, UNVA, Spectacle Independence
		11	NR	58.8 ± 5.3	trifocal-AT LISAtri 839MP	AT LISA tri 839MP IOL	diffractive IOL	C		
18	Bala, C. (2022) [44]	149	60.26%	70.5 ± 4.3	EDOF IOL	DFT015 (EDOF) IOL	nondiffractive IOL	G	6 m	UDVA, CDVA, UIVA, CIVA, UNVA, CNVA, SE, CS under photopic condition, CS under mesopic condition, halo, glare, Spectacle Independence
		117	54.17%	70.0 ± 3.9	monofocal IOL	monofocal IOL SN60WF	aspheric IOL	A		
19	Chang, D. H. (2022) [45]	147	61.49%	68.0 ± 7.5	EDOF IOL	TECNIS Symfony (EDOF)	refractive and diffractive IOL	G	6 m	UDVA, CDVA, UIVA, CIVA, UNVA, CNVA, SE, halo, glare, Spectacle Independence
		148	56.95%	67.9 ± 7.9	monofocal IOL	TECNIS Monofocal	aspheric IOL	A		
20	Ferreira, T. B. (2022) [46]	30	NR	62.6 ± 6.7	trifocal-other new trifocal IOLs	Tecnis Synergy(Vision)	wavefront-designed anterior aspheric surface	F	3 m	UDVA, CDVA, UIVA, CIVA, UNVA, CNVA, SE, halo, glare, Spectacle Independence
		30	NR	61.1 ± 6.0	trifocal-AcrySof IQ PanOptix TFNT00	Acrysof PanOptix TFNT00	diffractive IOLs	E		
		30	NR	60.1 ± 6.8	trifocal-FineVision POD F	POD F (Finevision)	diffractive IOLs	D		
21	Garzón, N. (2022) [47]	30	77.60%	75.87 ± 5.97	enhanced monofocal IOL	Eyhance model ICB00	refractive IOL	H	1 m	UDVA, CDVA, SE, CS under photopic condition
		30	62.90%	70.62 ± 8.11	monofocal IOL	monofocal ZCB00 IOL	aspheric IOL	A		
22	Gil, MÁ (2022) [48]	19	57.89%	74.3 ± 7.5	bifocal IOL	SVT250 Bifocal	diffractive and refractive IOL	B	6 m	CS under photopic condition, CS under mesopic condition, halo, Spectacle Independence
		20	75.00%	68.9 ± 12.9	bifocal IOL	ZKB00 Bifocal	diffractive IOL	B		
		19	78.95%	68.7 ± 10.3	trifocal-AT LISAtri 839MP	ATLISA Tri 839MP Trifocal	diffractive IOL	C		
		20	65.00%	73.3 ± 4.6	bifocal IOL	ZLB00 Bifocal	diffractive IOL	B		
		18	77.78%	71.6 ± 7.1	bifocal IOL	ATLISA 809 M Bifocal	diffractive IOL	B		
		20	75.00%	68.2 ± 6.2	EDOF IOL	Symfony ZXR00 Extended Depth of Focus	refractive and diffractive IOL	G		
		36	58.33%	72.1 ± 5.8	monofocal IOL	ZA9003 Monofocal	aspheric IOL	A		
23	Guarro, M. (2022) [49]	22	50.00%	71.00 ± 9.27	EDOF IOL	AcrySof IQ Vivity(Vivity)	nondiffractive IOL	G	3 m	SE, halo, glare,
		22	54.55%	73.52 ± 6.64	EDOF IOL	AT LARA 829MO(AT LARA)	diffractive IOL	G		
		22	54.55%	72.09 ± 4.45	EDOF IOL	TECNIS Symfony ZXR00(Symfony)	refractive and diffractive IOL	G		
		22	54.55%	76.19 ± 7.17	monofocal IOL	monofocal AcrySof IQ SN60WF	aspheric IOL	A		
24	McCabe, C. (2022) [50]	106	55.14%	68.8 ± 7.82	EDOF IOL	DFT015 (EDOF) IOL	nondiffractive IOL	G	6 m	UDVA, CDVA, UIVA, CIVA, UNVA, CNVA, SE, CS under mesopic condition, halo, glare, Spectacle Independence
		111	56.63%	68.8 ± 6.63	monofocal IOL	monofocal IOL SN60WF	aspheric IOL	A		
25	Nanavaty, M. A. (2022) [51]	25	NR	72.79 ± 8.04	enhanced monofocal IOL	TECNIS Eyhance IOL (ICB00)	refractive IOL	H	1 m,3–6 m	UDVA, CDVA, UIVA, CIVA, SE
		25	NR	77.16 ± 9.79	monofocal IOL	monofocal RayOne	aspheric IOL	A		
26	Pantanelli, S. M. (2022) [52]	24	83.30%	68.45 ± 6.53	EDOF IOL	Alcon AcrySof Vivity(EDOF)	nondiffractive IOL	G	3 m	UDVA, CDVA, UIVA, CIVA, UNVA, CNVA, SE, halo, glare, Spectacle Independence
		27	70.40%	68.34 ± 6.48	monofocal IOL	(Bausch & Lomb enVista) intraocular lenses	aspheric IOL	A		
27	Sevik, M. O. (2022) [53]	15	46.67%	63.43 ± 7.11	bifocal IOL	bifocal Tecnis + 2.75 D (ZKB00)	diffractive IOL	B	1 m,3 m,6 m	UDVA, UIVA, UNVA, SE, CS under mesopic condition, halo, glare, Spectacle Independence
		14	42.86%	63.96 ± 8.42	EDOF IOL	Tecnis Symfony (ZXR00) EDOF	refractive and diffractive IOL	G		
28	Asena, L. (2023) [54]	26	NR	68.92 ± 9.87	EDOF IOL	AcrySof®IQ Vivity EDOF	nondiffractive IOL	G	3 m	UDVA, CDVA, UIVA, CIVA, UNVA, SE, CS under photopic condition, CS under mesopic condition,
		26	NR	63.06 ± 9.72	trifocal-AcrySof IQ PanOptix TFNT00	AcrySof®IQ PanOptix	diffractive IOL	E		

### Primary and secondary outcomes

The primary outcomes included corrected and uncorrected binocular visual acuities (distant, intermediate and near visual acuity), spectacle independence (distant, intermediate and near spectacle independence). The secondary comprehensive endpoints included optical quality (holas and glare) and contrast sensitivity (under photopic and mesopic conditions). Primary outcomes are used to evaluate validity and secondary outcomes are used to evaluate safety.

### Statistical analysis

The mean difference (MD) and its 95% confidence interval (CI) were used to evaluate the effects of the outcomes for continuous variables, and the odds ratio (OR) and its 95% confidence interval (CI) were used to evaluate the effects of the outcomes for dichotomous variable. The entire analysis was performed using the random effects model. Conducting network meta-analyses within Stata software (version 17.0), we executed data processing, generated network evidence plots, performed an inconsistency test, ranked interventions using the Surface Under the Cumulative Ranking (SUCRA), created forest plots, and analyzed funnel plots. Network evidence plots visually depicted treatment comparisons for each outcome, and the inconsistency test evaluated metric coherence (with *p* < 0.05 indicating significant inconsistency). It’s crucial to emphasize that SUCRA is integral to these analyses. SUCRA ranks intervention efficacy based on cumulative probability, with higher values (closer to 1) signifying greater effectiveness. Serving as a valuable tool, SUCRA summarizes and interprets relative intervention efficacy across diverse outcomes in network meta-analyses. Forest plots aided in pairwise comparisons (a result of 0 denoting no significant difference), and potential publication bias was explored through a funnel plot.

## Results

### Characteristics of the included studies

**Fig. 1:**
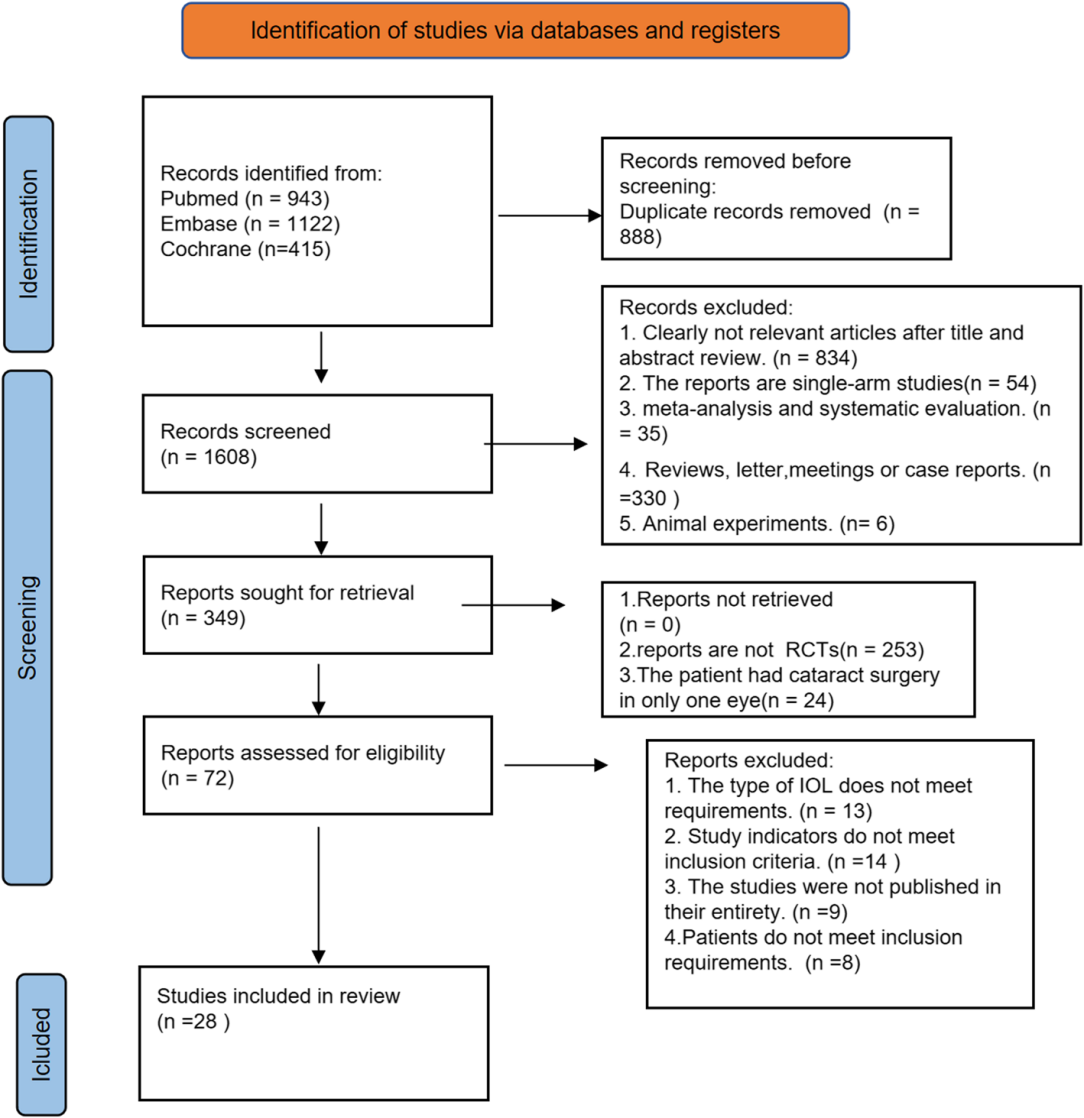
Flow diagram of literature search

Initially, a total of 2465 patients were retrieved from three databases, by screening out duplicates and reading titles and abstracts to screen out non-compliant literature, and after careful reading and screening, eventually a total of 28 RCTs reporting 2580 patients (5160 eyes) with 8 interventions [27–41, 55], the detailed literature screening process shown in Fig. 1. Patients are followed up for no less than 1 month after surgery on both eyes, the 8 interventions implanted eight different types of IOLs in both eyes. We classified IOLs into the following types: monofocal, bifocal, trifocal, EDOF, enhanced monofocal IOLs. Among these IOLs, trifocal IOLs have been more studied and categorized, so they are categorized into four categories, including AT LISAtri 839MP, FineVision POD F, AcrySof IQ PanOptix and other new trifocal IOLs. Table 1 shows the characteristics of all included studies.

### Visual acuity

#### Uncorrected near visual acuity (UNVA)

**Fig. 2:**
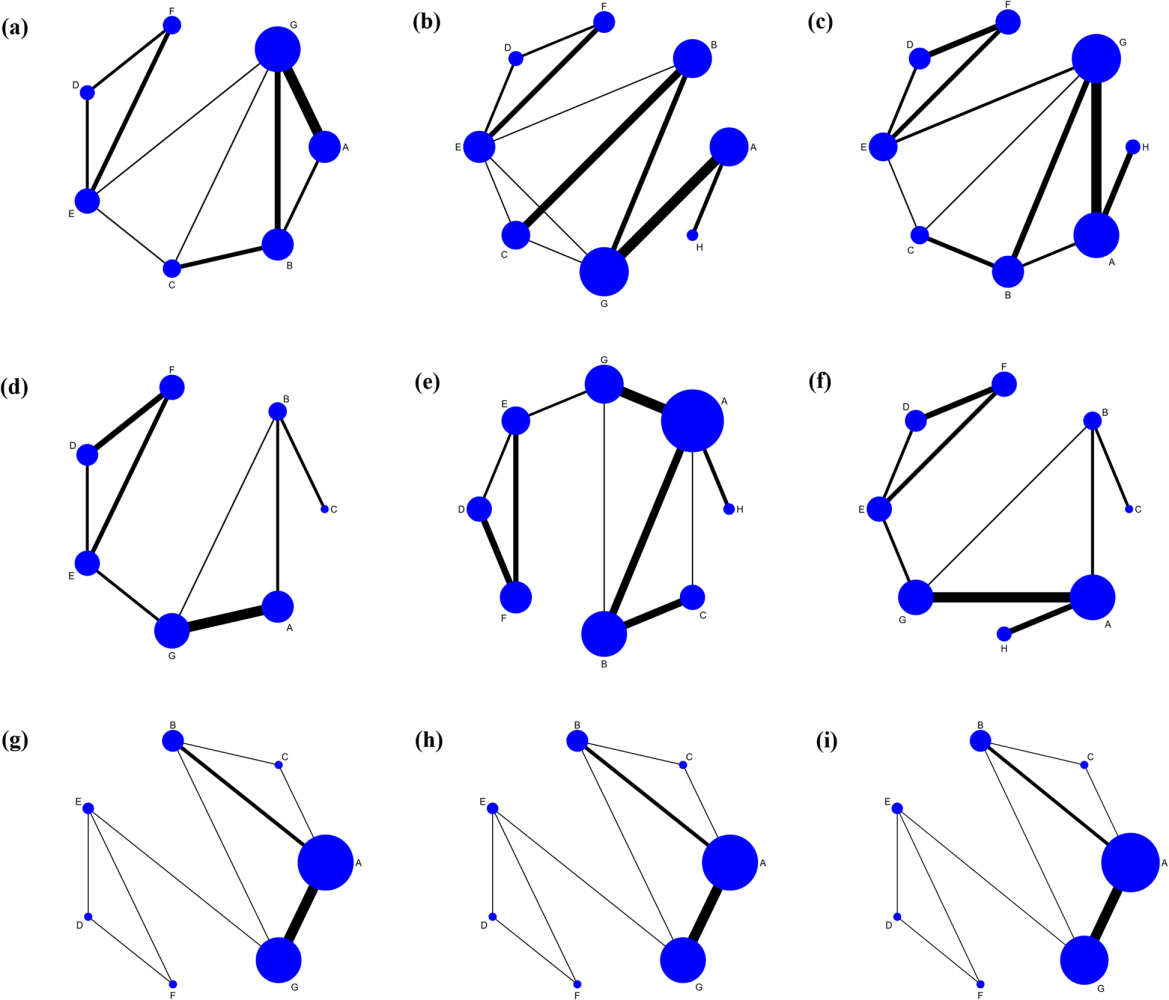
Network graph of primary indicators. Note: **A**: standard monofocal IOLs group, **B**: bifocal IOLs group, **C**: AT LISAtri 839MP IOLs group, **D**: FineVision POD F IOLs group, **E**: AcrySof IQ PanOptix IOLs group, **F**: other new trifocal IOLs group, **G**: EDOF IOLs group, **H**: enhanced monofocal IOLs group; (**a**): Network graph of UNVA, (**b**): Network graph of UIVA, (**c**): Network graph of UDVA, (**d**): Network graph of CNVA, (**e**): Network graph of CIVA, (**f**): Network graph of CDVA, (**g**): Network graph of distant spectacle independence, (**h**): Network graph of intermediate spectacle independence, (**i**) Network graph of near spectacle independence

**Fig. 3:**
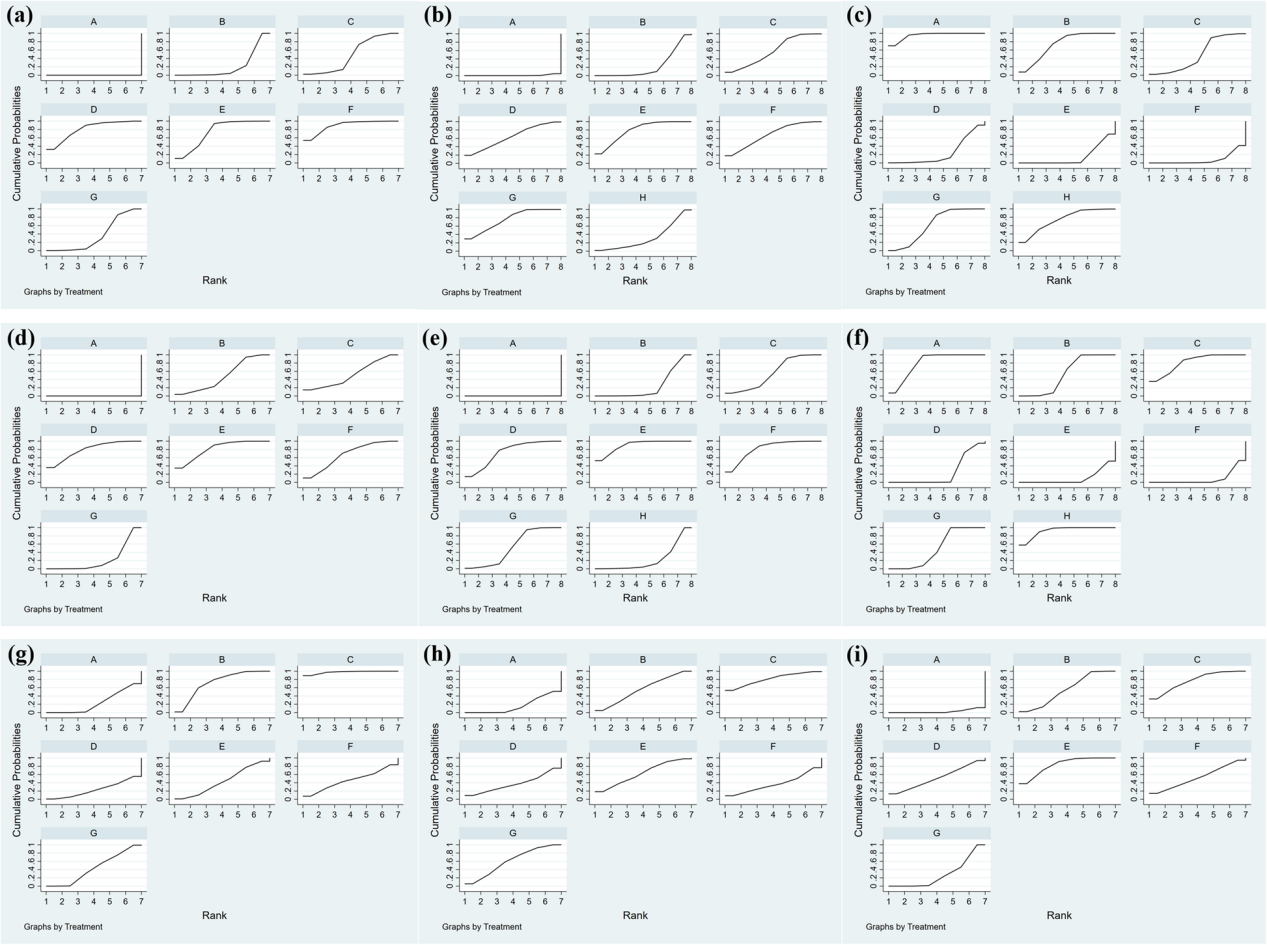
SUCRA of primary indicators. Note: A: standard monofocal IOLs group, B: bifocal IOLs group, C: AT LISAtri 839MP IOLs group, D: FineVision POD F IOLs group, E: AcrySof IQ PanOptix IOLs group, F: other new trifocal IOLs group, G: EDOF IOLs group, H: enhanced monofocal IOLs group; (a): SUCRA of UNVA, (b): SUCRA of UIVA, (c): SUCRA of UDVA, (d): SUCRA of CNVA, (e): SUCRA of CIVA, (f): SUCRA of CDVA, (g): SUCRA of distant spectacle independence, (h): SUCRA of intermediate spectacle independence, (i)SUCRA of near spectacle independence

**Fig. 4:**
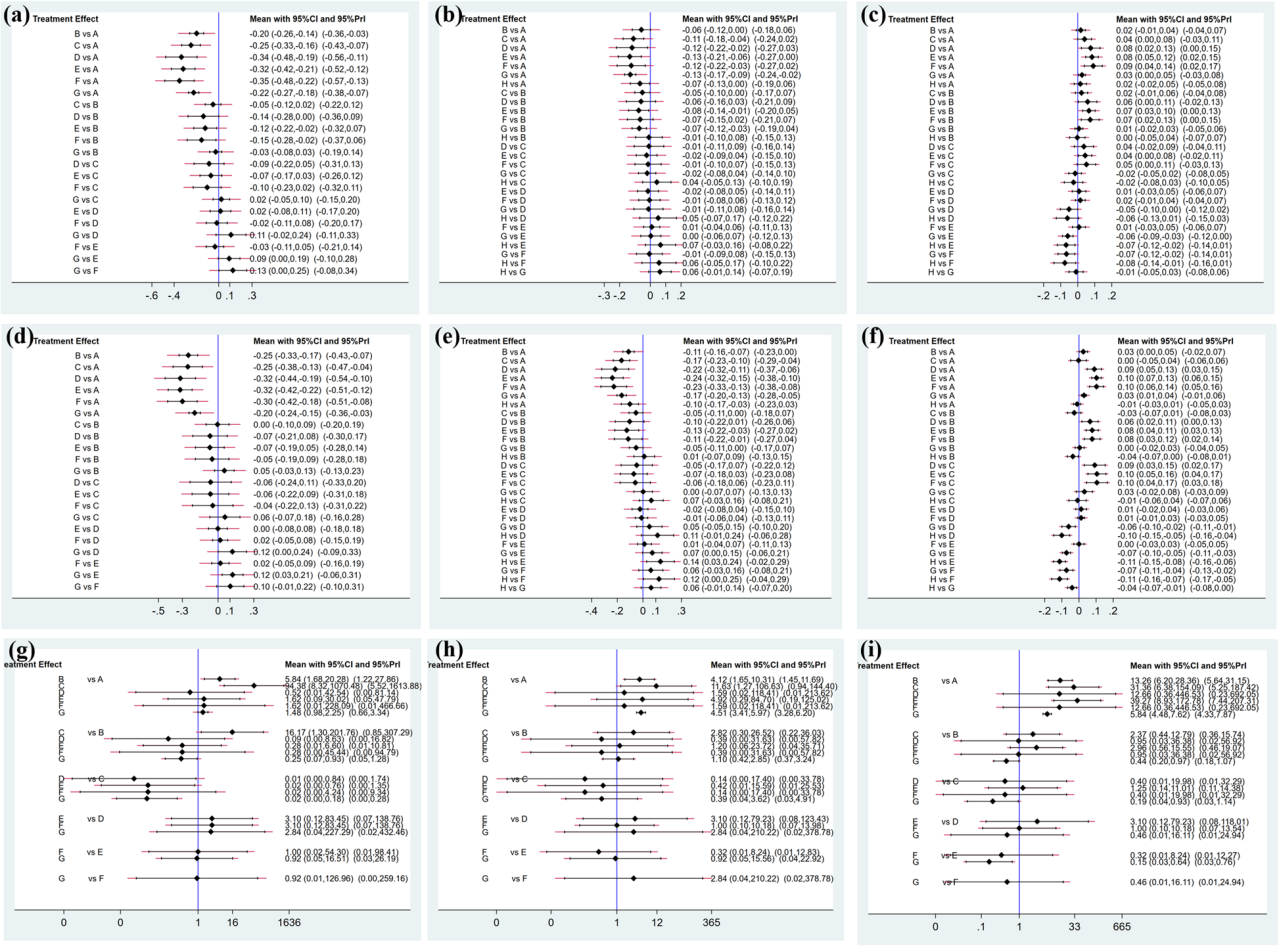
Forest plot of primary indicators. Note: **A**: standard monofocal IOLs group, **B**: bifocal IOLs group, **C**: AT LISAtri 839MP IOLs group, **D**: FineVision POD F IOLs group, **E**: AcrySof IQ PanOptix IOLs group, **F**: other new trifocal IOLs group, **G**: extended depth-of-focus(EDOF) IOLs group, **H**: enhanced monofocal IOLs group; (**a**): Forest plot of UNVA, (**b**): Forest plot of UIVA, (**c**): Forest plot of UDVA, (**d**): Forest plot of CNVA, (**e**): Forest plot of CIVA, (**f**): Forest plot of CDVA, (**g**): Forest plot of distant spectacle independence, (**h**): Forest plot of intermediate spectacle independence, (**i**): Forest plot of near spectacle independence

The network of UNVA included 18 studies involving 4 closed loop with 6 types of IOLs (Fig. 2a). In the comparison of the area under the curve, four groups trifocal IOLs ranked in the top four and other new trifocal IOLs group had the largest SUCRA (89.1%) (Fig. 3a). The 6 types of IOLs were significantly more effective than monofocal IOLs, among which, the trifocal IOLs were the most effective, and they inclued AT LISAtri 839MP (MD -0.25, 95% Cl -0.3, -0.16), FineVision POD F (MD -0.34, 95% Cl -0.48, -0.19), AcrySof IQ PanOptix (MD -0.32, 95% Cl: -0.42, -0.21) and other new trifocal IOLs (MD -0.35, 95% Cl:-0.48, -0.22). Also, AcrySof IQ PanOptix(MD -0.12, 95% Cl:-0.22, -0.02) and other new trifocal IOLs(MD -0.15, 95% Cl:-0.28, -0.02) have better UNVA than bifocal IOLs (Fig. 4a).

For UIVA, there were 19 studies and four closed loops in evidence network (Fig. 2b). AcrySof IQ PanOptix IOLs had the largest SUCRA (78.4%), followed by EDOF IOLs (SUCRA 75.8%), and the SUCRA (32.4%) of enhanced monofocal IOLs were better than monofocal IOLs and bifocal IOLs (Fig. 3b). This result demonstrates the superiority of these two IOLs in vision at intermediate distances. For the pairwise meta-analysis, AT LISAtri 839MP IOLs (MD -0.11, 95% Cl -0.18, -0.04), FineVision POD F IOLs (MD -0.12, 95% Cl -0.22, -0.04), AcrySof IQ PanOptix IOLs (MD -0.13, 95% Cl -0.21, -0.06), other new trifocal IOLs (MD -0.12, 95% Cl -0.22, -0.03) and EDOF IOLs (MD -0.13, 95% Cl -0.17, -0.09) were more significantly effective than monofocal IOLs according to the results of the forest plot. AcrySof IQ PanOptix IOLs and EDOF IOLs also showed the effectiveness when compared with bifocal IOLs. The other IOL types did not show significant differences (Fig. 4b).

#### Uncorrected distant visual acuity (UDVA)

There were 23 studies reported the UDVA, there were the most studies on monofocal IOLs and EDOF IOLs according to evidence network (Fig. 2c). In a comparison of the SUCRA, the monofocal IOLs has the largest area (SUCRA 95.1%), indicating that the monofocal IOLs provides a better UDVA for the patient, the results for the SUCRA are presented in (Fig. 3c). For the pairwise meta-analysis, monofocal IOLs group was more significantly effective compared to FineVision POD F IOLs (MD 0.08, 95% Cl 0.02–0.13), AcrySof IQ PanOptix IOLs (MD 0.08, 95% Cl 0.05–0.12) and other new trifocal IOLs group (MD 0.09, 95% Cl 0.04–0.14), which were all trifocal IOLs. Besides, bifocal, EDOF, and the enhanced monofocal IOLs were better than AcrySof IQ PanOptix IOLs and other new trifocal IOLs (Fig. 4c).

#### Corrected visual acuity

The corrected visual acuities yielded a tendency similar to that seen in the uncorrected outcomes. Distant and near visual acuity formed two closed loops and intermediate visual acuity formed three according to the evidence network (Fig. 2d-f). The sorting results of SUCRA for all four groups of trifocal IOLs were in the top four, with AcrySof IQ PanOptix IOLs having the maximum value both in corrected near (SUCRA 81.2%) visual acuity (CNVA) and corrected intermediate (SUCRA 89.9%) visual acuity (CIVA) (Fig. 3d, e). In pairwise comparison, all multifocal IOLs were more effective than monofocal IOLs in providing improved vision at intermediate and near distances. Of these, AcrySof IQ PanOptix IOLs is most effective when compared to monofocal IOLs at near and intermediate distances (Fig. 4d, e). As for corrected distant visual acuity (CDVA) we found that enhanced monofocal IOLs had the highest SUCRA (92.3%) by ranking the effects of all interventions (Fig. 3f). And FineVision POD F, AcrySof IQ PanOptix and other new trifocal IOLs groups were more effective than other groups (Fig. 4f).

### Spectacle independence

There were 11 studies reported spectacle independence. We studied spectacle independence separately for distant, intermediate and near distances. All three indicators form three closed loops according to the network evidence (Fig. 2g-i). Ranking the effects of all interventions by SUCRA probability, we found that AT LISAtri 839MP IOLs had the highest spectacle in distant (SUCRA 97.5%) and intermediate SUCRA (80.7%) spectacle independence, AcrySof IQ PanOptix IOLs (SUCRA 83.0%) was the top and AT LISAtri 839MP IOLs (SUCRA 62.6%) was the second in near spectacle independence (Fig. 3g-i). For the pairwise meta-analysis, AT LISAtri 839MP IOLs significantly improved distant spectacle independence compared to monofocal, bifocal, FineVision POD F, AcrySof IQ PanOptix and EDOF IOLs (Fig. 4g); Bifocal, AT LISAtri 839MP and EDOF IOLs significantly improved spectacle independence at intermediate distances compared to monofocal IOLs (Fig. 4h); As for in near spectacle independence, when bifocal (OR 13.26, 95% Cl 6.20, 28.36), AT LISAtri 839MP (OR 31.6, 95% Cl 6.38, 154.09), AcrySof IQ PanOptix (OR 39.27, 95% Cl 8.93, 172.78) and EDOF IOLs (OR 5.84, 95% Cl 4.48, 7.62) were compared with monofocal IOLs, the results were significantly valid (Fig. 4i).

### Contrast sensitivity (CS)

We reported the result of contrast sensitivity under both photopic and mesopic conditions and collected data at spatial frequencies of 3, 6, 12, and 18 cycles per degree (cpd). Their network relationship is shown in Supplementary Fig. 1 (a-h). Ranking the effectiveness of all IOLs by SUCRA results, at the four spatial frequencies, the SUCRA values for monofocal and enhanced monofocal IOLs are consistently in the top, the values for bifocal and AT LISAtri 839MP IOLs are consistently in the bottom under both photopic and mesopic conditions, besides, the contrast sensitivity of FineVision POD F IOLs group gradually improves with increasing spatial frequency (Supplementary Fig. 2a-h). Under photopic condition, when a comparison is made between two IOLs, monofocal and enhanced monofocal IOLs showed better CS than bifocal IOLs and AT LISAtri 839MP IOLs at the four spatial frequencies (Supplementary Fig. 3a-d). At spatial frequencies of 12 cpd, FineVision POD F IOLs were more effective than bifocal (MD 0.20, 95% Cl 0.02, 0.39) and AT LISAtri 839MP IOLs (MD 0.21, 95% Cl 0.04, 0.38) (Supplementary Fig. 3c). At spatial frequencies of 18 cpd, FineVision POD F IOLs were more effective than AT LISAtri 839MP IOLs (Supplementary Fig. 3d). Under mesopic condition, monofocal and enhanced monofocal IOLs more significantiy effective than bifocal, AT LISAtri 839MP, AcrySof IQ PanOptix, other new trifocal and EDOF IOLs at spatial frequencies of 3 cpd (Supplementary Fig. 3e) and 6 cpd (Supplementary Fig. 3f). CS of some multifocal IOLs becomes progressively higher with increasing spatial frequency (Supplementary Fig. 3g, h), especialiy FineVision POD F IOLs were more effective than other groups at spatial frequencies of 18 cpd (Supplementary Fig. 3h).

### Halos and glare

Halos and glare are two of the most common visual impairments that patients experience after multifocal IOL surgery. The network evidence showed a total of 7 closed loops for halos and 4 closed loops for glare (Supplementary Fig. 1i, j). As for halos, monofocal (SUCRA 91.6%) and enhanced monofocal IOLs (SUCRA 86.0%) are the best according to SUCRA, and the four groups of trifocal IOLs are the worst (Supplementary Fig. 2i). Bifocal (OR 4.02, 95% Cl 1.39, 11.58), AT LISAtri 839MP (OR 8.31, 95% Cl 1.85, 37.22), AcrySof IQ PanOptix (OR 10.98, 95% Cl 1.31, 91.99) and EDOF IOLs (MD 4.94, 95% Cl 2.17, 11.25) more likely to had halos than monofocal IOLs in the pairwise meta-analysis (Supplementary Fig. 3i). Besides, in the ordering of the areas under the curve, other new trifocal IOLs (SUCRA 71.3%) and AT LISAtri 839MP IOLs (SUCRA 68.9%) rank in the first two in glare (Supplementary Fig. 2j). For the pairwise meta-analysis, none of the results of the comparisons between the various interventions were significantly different from each other (Supplementary Fig. 3t).

### Publication bias and quality assessment

**Fig. 5:**
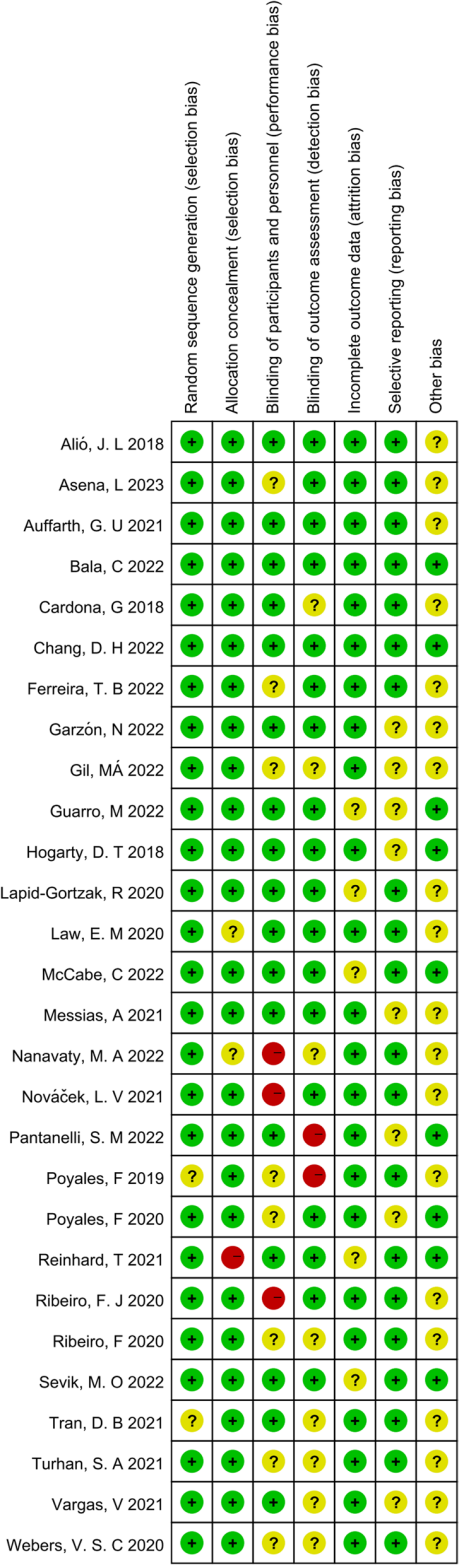
Risk of bias summary

The funnel plot of this study revealed that most of the scatter points were located on both sides of the vertical line. They were basically symmetrical and may have had a certain degree of publication bias. The funnel plot of the effect on visual outcome after implantation of eight types of artificial lenses in both eyes is presented in Supplementary Fig. 4 (a-i) and Supplementary Fig. 5 (a-j). Based on risk of bias assessment, there were two randomized clinical trials showed a low high of bias due to inadequate sequence generation. Regarding allocation concealment, one trial had a high risk and two trial did not mention. Three trials were not blinded to participants and personnel, while two were not blinded to outcome assessment. In all randomized clinical studies, the likelihood of selective reporting bias and incomplete outcome data was minimal. In all included trials, other biases mostly unclear. Therefore, the overall quality of the incorporated articles is high, respectively, Fig. 5 and Supplementary Fig. 6 show the risk-of-bias summary and risk-of-bias graph for selected studies. The results of the consistency analysis across the study indicators can be seen in Table 2, in performing the consistency test, we found that UNVA and CNVA were less consistent, the other indicators were more consistent, and there were no significant differences between direct and indirect comparisons. The League tables are shown as mean and 95% CIs to assess whether there was a signifcant diference in the efcacy or safety of each regimen (Supplementary Table 1).

**Table 2:** Heterogeneity test form

Targets	*p*
UDVA	*P* = 0.2737
UNVA	*P* = 0.0002
UIVA	*P* = 0.1106
CDVA	*P* = 0.6571
CNVA	*P* = 0.0263
CIVA	*P* = 0.8018
SE	*P* = 0.1863
3 cpd under photopic	*P* = 0.1685
6 cpd under photopic	*P* = 0.0561
12 cpd under photopic	*P* = 0.4358
18 cpd under photopic	*P* = 0.6118
3 cpd under mesopic	*P* = 0.6311
6 cpd under mesopic	*P* = 0.8765
12 cpd under mesopic	*P* = 0.2417
18 cpd under mesopic	*P* = 0.1199
Distant Spectacle Independence	*P* = 0.0541
Intermediate Spectacle Independence	*P* = 0.0618
Near Spectacle Independence	*P* = 0.9844
Halos	*P* = 0.9362
Glare	*P* = 0.9628

## Discussion

This study represents the first attempt to comprehensively compare the efficacy and safety of different IOLs for presbyopia-correcting cataract surgery using a network meta-analysis based on a frequency-based framework. The primary objective was to provide a credible evidence-based medical foundation for choosing clinical IOLs. The results of our network meta-analysis are promising. The evaluation focused on effectiveness, considering visual acuity and spectacle independence, as well as safety, assessed based on optical quality. Multifocal IOLs, particularly trifocal IOLs (AcrySof IQ PanOptix TFNT00 and FineVision POD FT IOLs), prove effective in addressing patients’ vision needs at intermediate and near distances, enhancing presbyopia treatment and spectacle independence. However, trifocal IOLs are associated with a reduction in optical quality, manifesting as decreased contrast sensitivity, halos, and glare. EDOF IOLs demonstrate comparable effectiveness to trifocal IOLs in improving postoperative intermediate visual acuity. In terms of optical quality, EDOF IOLs exhibit higher contrast sensitivity than trifocal IOLs at lower spatial frequencies, with a lower likelihood of halos occurring. Enhanced monofocal IOLs maintain the excellent distant visual acuity and quality of traditional monofocal IOLs while enhancing UIVA and CIVA. However, indirect comparisons suggest that enhanced monofocal IOLs may not match the intermediate visual acuity of trifocal and EDOF IOLs but are comparable to bifocal IOLs. In conclusion, our findings underscore the advantages and potential of trifocal, EDOF, and enhanced monofocal IOLs in enhancing visual acuity for presbyopia-correcting cataract surgery.

Initially, we assessed the efficacy of different IOLs. For binocular UNVA and CNVA, statistical differences were observed when comparing multifocal and EDOF IOLs to monofocal IOLs, with trifocal IOLs demonstrating the best near visual acuity, consistent with previous research [56]. A comparison between AT LISA tri 839MP and EDOF IOLs in near vision revealed no significant difference, contrary to another study, possibly due to our inclusion of four groups of trifocal IOLs with different designs [36]. Trifocal IOLs ensure vision at three distances through light distribution, with more light distribution resulting in better vision [57]. All four trifocal IOLs groups had distinct light distributions at near distances, with AT LISA tri 839MP IOLs, a conventional trifocal IOLs, having only 30% light distribution at near distances [57]. The ranking of UNVA area under the curve revealed the largest share for the other new trifocal IOLs. In a comparison with Acrysof PanOptix, the new trifocal IOLs displayed extensive vision, particularly at near distances, and superior mesopic performance in cataract surgery patients [58]. Regarding binocular UIVA and CIVA, a Cochrane database evaluation from 2020 found that trifocal IOLs implantation resulted in better UIVA at one year, without a significant advantage for uncorrected near and distance vision [59]. A subsequent 2023 evaluation, expanded in patient age range, confirmed these findings [10]. Additionally, EDOF IOLs, with an achromatic diffractive surface provide a low add foci, extending the range of vision from distance through intermediate, a study with a three-month follow-up, found EDOF IOLs to be a well-tolerated option for correcting far and intermediate vision [60]. In our study, compared to most trifocal IOL groups, EDOF IOLs exhibited better UIVA, but trifocal IOLs showed a more significant advantage in CIVA. Enhanced monofocal IOLs, a recent introduction, demonstrated advantages in intermediate distance vision. However, compared to EDOF IOLs, the latter showed superior near vision outcomes with a higher rate of spectacle independence [24]. Monofocal IOLs are designed for clear vision at the retinal plane, providing detailed and high-contrast vision for distant objects [61]. Our results also indicate that other IOL types were not as effective as standard monofocal and enhanced monofocal IOLs for binocular UDVA and CDVA.

Postoperative changes in refractive power often necessitate customized fine-tuning to optimize spectacle independence for patients [62]. In our literature review, there was no mention of spectacle independence for enhanced monofocal IOLs. Therefore, we compared seven types of IOLs for this indicator, finding that other IOLs exhibited superior spectacle independence compared to monofocal IOLs at both intermediate and near distances. At intermediate distances, there was no significant difference between EDOF and trifocal IOLs. However, at near distances, trifocal IOLs demonstrated more pronounced spectacle independence. A clinical report by Schallhorn on cataract patients indicated that EDOF IOLs achieved 83.6% spectacle independence for near vision and 95.4% for distance vision [63]. Another study comparing presbyopia-correcting IOLs reported that 71–96% of patients achieved spectacle independence, with the EDOF group showing a lower percentage than the trifocal group [64]. In our spectacle independence ranking, AT LISAtri 839MP IOLs secured the top position at all three distances. AcrySof IQ PanOptix IOLs claimed the second spot for intermediate and near spectacle independence, while the EDOF IOLs group ranked third for spectacle independence at intermediate distances. Rita Mencucci’s study on AT LISAtri 839MP IOLs reported that 33% of trifocal IOL patients and 40% of EDOF IOL patients needed reading glasses for some activities, aligning with our findings [65].

While our current study suggests that trifocal IOLs are the most effective, this conclusion may not be definitive. Multifocal IOLs were designed to split light into different focal points. However, the neuro-adaptive process required to process these different images can be time-consuming and frustrating for patient [66]. Therefore, we examined the safety of various IOLs in terms of optical quality, focusing on CS, halos, and glare. In terms of CS, significant decreases were observed in other IOL groups compared to monofocal and enhanced monofocal IOLs under both photopic and mesopic conditions, with bifocal and AT LISAtri 839MP IOLs exhibiting the most noticeable effects. The CS of both EDOF and FineVision POD F IOLs increased with frequency, and EDOF IOLs have better CS only at lower spatial frequencies, with no significant difference at higher spatial frequencies, in line with previous reports [67, 68]. This aligns with the findings of an RCT related to a multifocal IOL reported by Gil, MÁ in 2022 [69, 70]. Preethi Karuppiah’s report comparing trifocal and EDOF IOLs also indicated better CS in EDOF IOLs [70]. Regarding halos, the monofocal and enhanced monofocal IOLs group exhibited fewer halos, with no statistically significant differences among other high-quality IOL groups. For glare, no statistical differences were found when comparing all groups, potentially due to the limited number of included studies. Multifocal IOLs are reported to have more pronounced optical phenomena such as halos and glare compared to monofocal IOLs in most studies [71, 72]. A meta-analysis by Sumitra in 2019 comparing multifocal IOLs and standard monofocal IOLs supported our study’s findings, indicating a greater risk of adverse visual phenomena with multifocal IOLs [56], A report by Leyla Asena found less optical interference and visual impairment in patients after bilateral implantation of EDOF IOLs than trifocal IOLs, and we supporting this result [73].

Posterior Capsular Opacification (PCO) is a common complication following cataract surgery and can impact visual outcomes by causing distortion and loss of corrected visual acuity. Clinically significant PCO requiring Nd: YAG capsulotomy adversely affects visual acuity. In Jorge’s study, during the 3, 6, and 12-month follow-ups, the ReSTOR group had 6 patients (17.6%) requiring Nd: YAG capsulotomy, and the bifocal AT LISA group had 3 patients (8.8%) at the 12-month follow-up only [27]. Another study by Bilbao-Calabuig suggested that AT Lisa 839 IOLs takes longer for PCO development compared to FineVision IOLs [74]. Decentration and tilt are also common complications of cataract surgery. They increase high-order aberrations and reduce visual quality. Different IOLs have varied effects on visual quality due to postoperative decentration and tilt. Aspheric and multifocal IOLs are more susceptible to decentration and tilt compared to other types of IOLs [75]. Research indicates that decentration and tilt have more pronounced effects on refractive multifocal IOLs than diffractive multifocal IOLs, and more pronounced effects on EDOF IOLs than refractive and diffractive multifocal IOLs [76]. The above complications are also common complications after cataract surgery, but due to the lack of data support, we did not conduct in-depth research, but this also provides a new direction for our future research.

Our study presents a comprehensive assessment of the effectiveness of different IOLs, making it the largest network meta-analysis of RCTs to date. The analysis covers the safety and efficacy of the most common IOLs across various healthcare institutions, offering valuable guidance for patients in selecting the most suitable IOLs and enhancing postoperative satisfaction. The study highlights the importance of analyzing individual preoperative conditions and overall physical status when choosing an IOL, taking into account financial considerations and lifestyle needs. However, out network meta-analysis has certain limitations. Firstly, the inclusion of 28 RCTs introduces variability due to the lack of detailed methodological information in some studies. Bias concerns related to randomization were addressed through bias analyses, and efforts were made to standardize study protocols. Secondly, discrepancies in measurements, criteria, and patient inclusion among different RCTs could contribute to bias. Inconsistent definitions of proximal and intermediate measurements were identified as a primary reason for bias in the assessment of uncorrected and corrected near and intermediate visual acuity. Thirdly, the relatively short follow-up times in the included RCTs and limited studies on postoperative complications, such as capsule fibrosis, YAG rates, and decentration, may affect the completeness of the analysis. Although you attempted to collect and analyze data on postoperative complications and patient satisfaction through questionnaires, variations in questionnaire topics and evaluation criteria hindered uniform categorization. Despite these limitations, our study contributes valuable insights into the field of cataract surgery and IOL selection, emphasizing the need for further research and individualized analysis of patient situations.

## Conclusion

In conclusion, the results of the network meta-analysis showed that various different IOLs were compared with each other and it was found that for patients with bilateral cataracts, binocular implantation of trifocal IOLs(especially AcrySof IQ PanOptix TFNT00 and FineVision POD FT IOLs) can give higher spectacle independence and good vision at intermediate and near distances, but need to overcome the decrease of optical quality, and EDOF and enhanced monofocal IOLs are also a good choices if there are more activities in daily life at intermediate distances. At the same time, enhanced monofocal IOLs are a better option for patients who are sensitive to decreased visual quality. However, more high-quality, large-sample, multicenter, randomized, double-blind trials are needed to confirm the reliability of the findings. The optimal treatment regimen should be determined on an individual patient basis, safety outcomes, and patient and caregiver decisions.

### Supplementary Information

**Supplementary Material 1.**

## Data Availability

All data generated or analyzed during this study are included in relevant published articles.

## Abbreviations

IOL: Intraocular lens
IOLs: Intraocular lenses
RCTs: Randomized Controlled Trials
EDOF: Extended Depth Of Focus
MD: Mean Difference
CI: Confidence Interval
OR: Odds Ratio
SUCRA: Surface under the cumulative ranking curve
UNVA: Uncorrected near visual acuity
UIVA: Uncorrected intermediate visual acuity
UDVA: Uncorrected distant visual acuity
CNVA: Corrected near visual acuity
CIVA: Corrected intermediate visual acuity
CDVA: Corrected distant visual acuity
CS: Contrast sensitivity
cpd: Cycles per degree
PCO: Posterior Capsular Opacification
