# Supplementary material for: Comparative efficacy and safety of all kinds of intraocular lenses in presbyopia-correcting cataract surgery: a systematic review and meta-analysis
Source: BMC Ophthalmol. 2024 Apr 16;24:172. doi: 10.1186/s12886-024-03446-1 (PMC11020619; doi:10.1186/s12886-024-03446-1)
Supplement: Supplementary file 1 — Supplementary Material 1. [file 12886_2024_3446_MOESM1_ESM.docx]

**
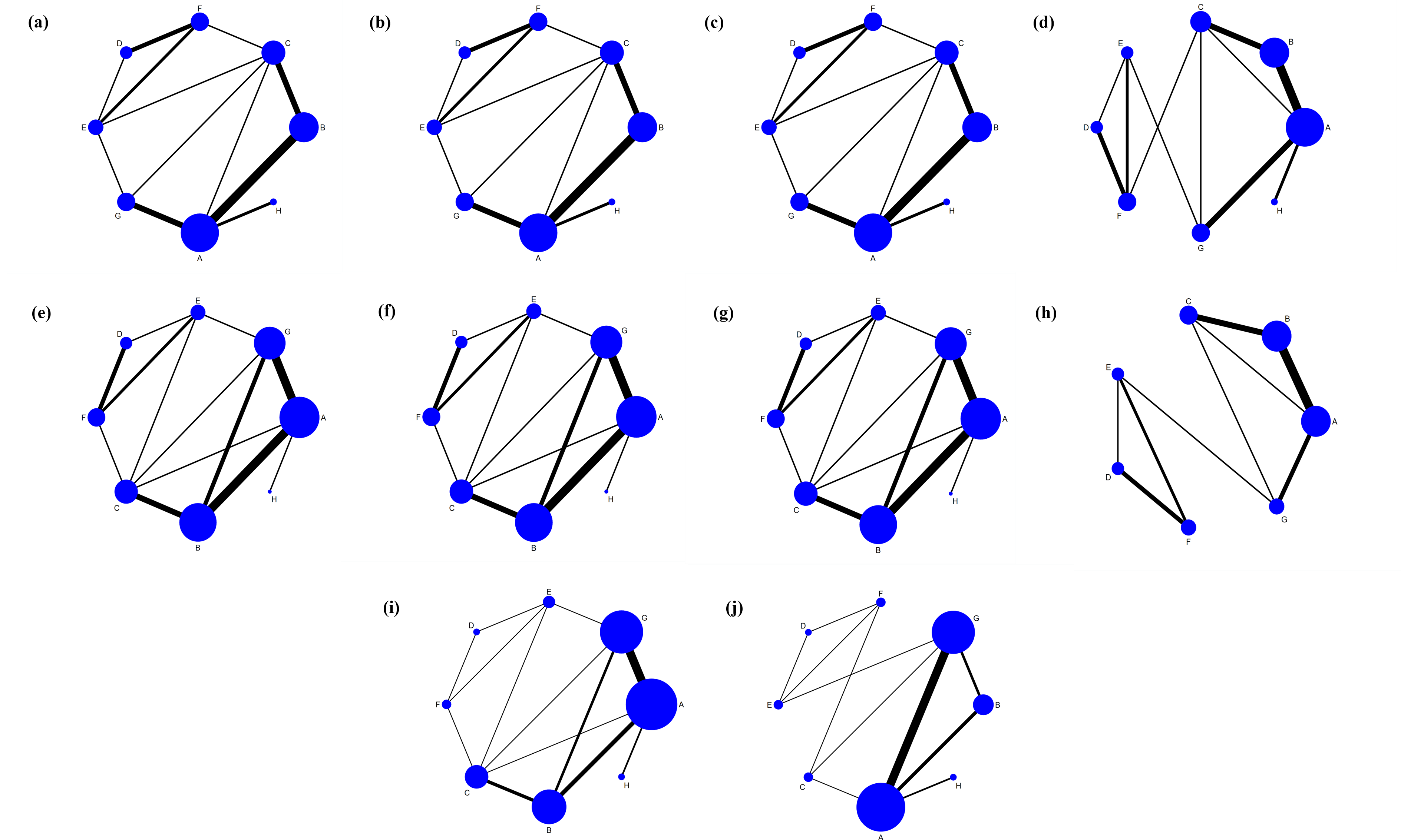
**

**Supplementary Figure 1.** Network of secondary indicators

Note:A: standard monofocal IOLs group, B: bifocal IOLs group, C: AT LISAtri 839MP IOLs group, D: FineVision POD F IOLs group, E: AcrySof IQ PanOptix IOLs group, F: other new trifocal IOLs group, G: extended depth-of-focus(EDOF) IOLs group, H: enhanced monofocal IOLs group; (a): Network graph of CS under photopic conditions at spatial frequencies of 3cpd, (b): Network graph of CS under photopic conditions at spatial frequencies of 6cpd, (c): Network graph of CS under photopic conditions at spatial frequencies of 12cpd, (d): Network graph of CS under photopic conditions at spatial frequencies of 18cpd, (e): Network graph of CS under mesopic conditions at spatial frequencies of 3cpd, (f): Network graph of CS under mesopic conditions at spatial frequencies of 6cpd, (g): Network graph of CS under mesopic conditions at spatial frequencies of 12cpd, (h): Network graph of CS under mesopic conditions at spatial frequencies of 18cpd, (i): Network graph of Halos, (j): Network graph of Glare


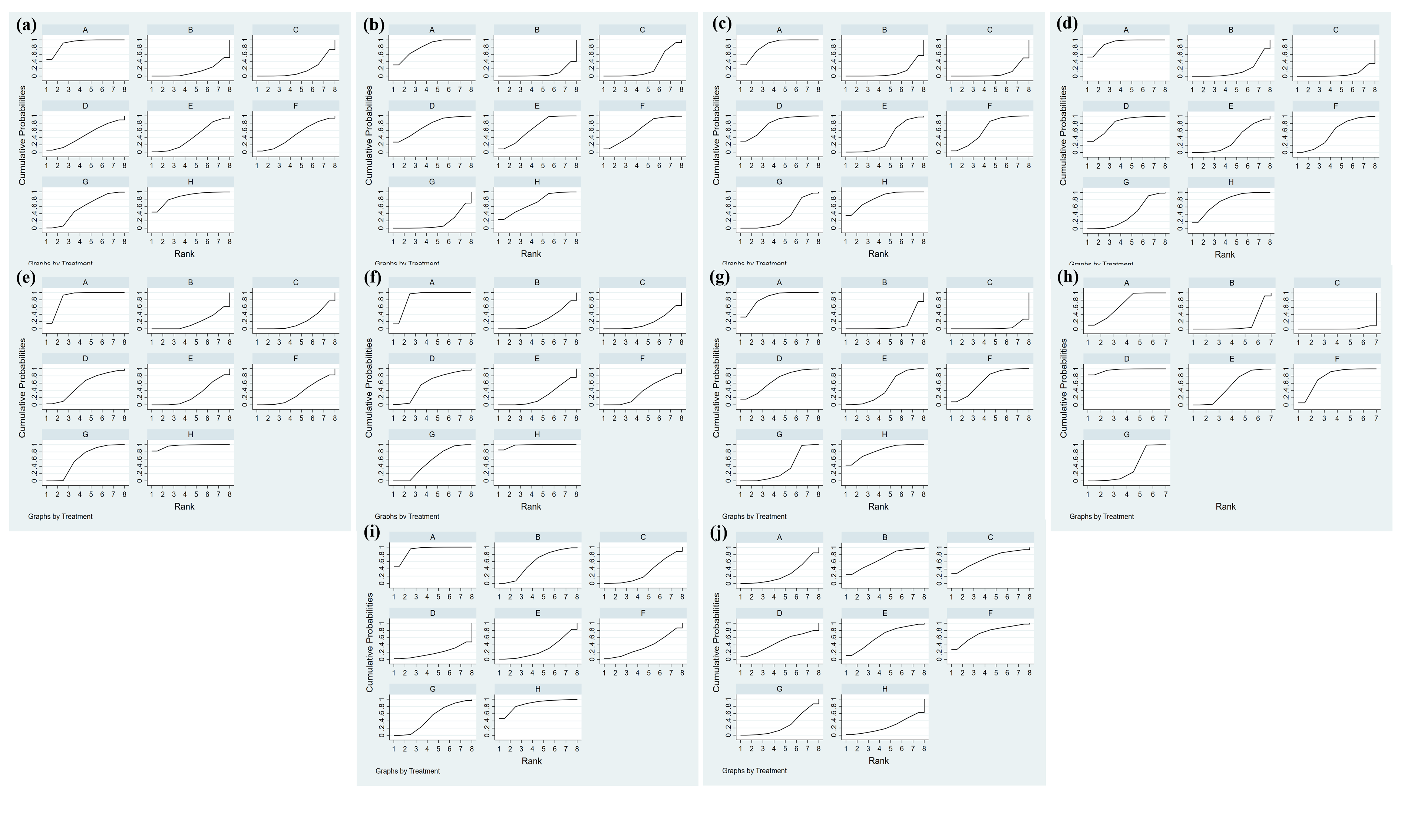


**Supplementary Figure 2.** SUCRA of secondary indicators

Note:A: standard monofocal IOLs group, B: bifocal IOLs group, C: AT LISAtri 839MP IOLs group, D: FineVision POD F IOLs group, E: AcrySof IQ PanOptix IOLs group, F: other new trifocal IOLs group, G: extended depth-of-focus(EDOF) IOLs group, H: enhanced monofocal IOLs group; (a): SUCRA of CS under photopic conditions at spatial frequencies of 3cpd, (b): SUCRA of CS under photopic conditions at spatial frequencies of 6cpd, (c): SUCRA of CS under photopic conditions at spatial frequencies of 12cpd, (d): SUCRA of CS under photopic conditions at spatial frequencies of 18cpd, (e): SUCRA of CS under mesopic conditions at spatial frequencies of 3cpd, (f): SUCRA of CS under mesopic conditions at spatial frequencies of 6cpd, (g): SUCRA of CS under mesopic conditions at spatial frequencies of 12cpd, (h): SUCRA of CS under mesopic conditions at spatial frequencies of 18cpd, (i): SUCRA of Halos, (j): SUCRA of Glare

**
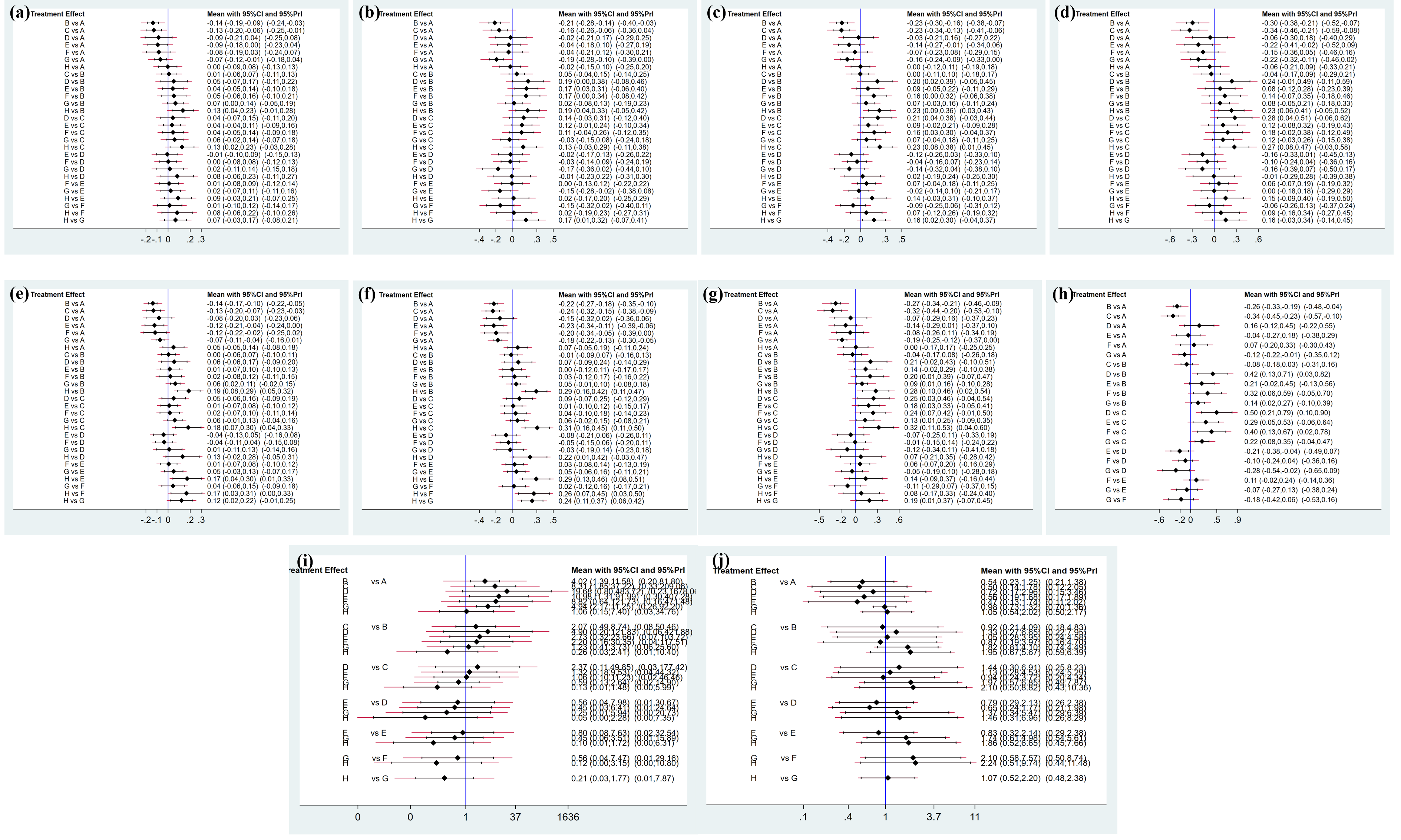
**

**Supplementary Figure 3.** Forest plot of secondary indicators

Note:A: standard monofocal IOLs group, B: bifocal IOLs group, C: AT LISAtri 839MP IOLs group, D: FineVision POD F IOLs group, E: AcrySof IQ PanOptix IOLs group, F: other new trifocal IOLs group, G: extended depth-of-focus(EDOF) IOLs group, H: enhanced monofocal IOLs group; (a): Forest plot of CS under photopic conditions at spatial frequencies of 3cpd, (b): Forest plot of CS under photopic conditions at spatial frequencies of 6cpd, (c): Forest plot of CS under photopic conditions at spatial frequencies of 12cpd, (d): Forest plot of CS under photopic conditions at spatial frequencies of 18cpd, (e): Forest plot of CS under mesopic conditions at spatial frequencies of 3cpd, (f): Forest plot of CS under mesopic conditions at spatial frequencies of 6cpd, (g): Forest plot of CS under mesopic conditions at spatial frequencies of 12cpd, (h): Forest plot of CS under mesopic conditions at spatial frequencies of 18cpd, (i): Forest plot of Halos, (j): Forest plot of Glare

**
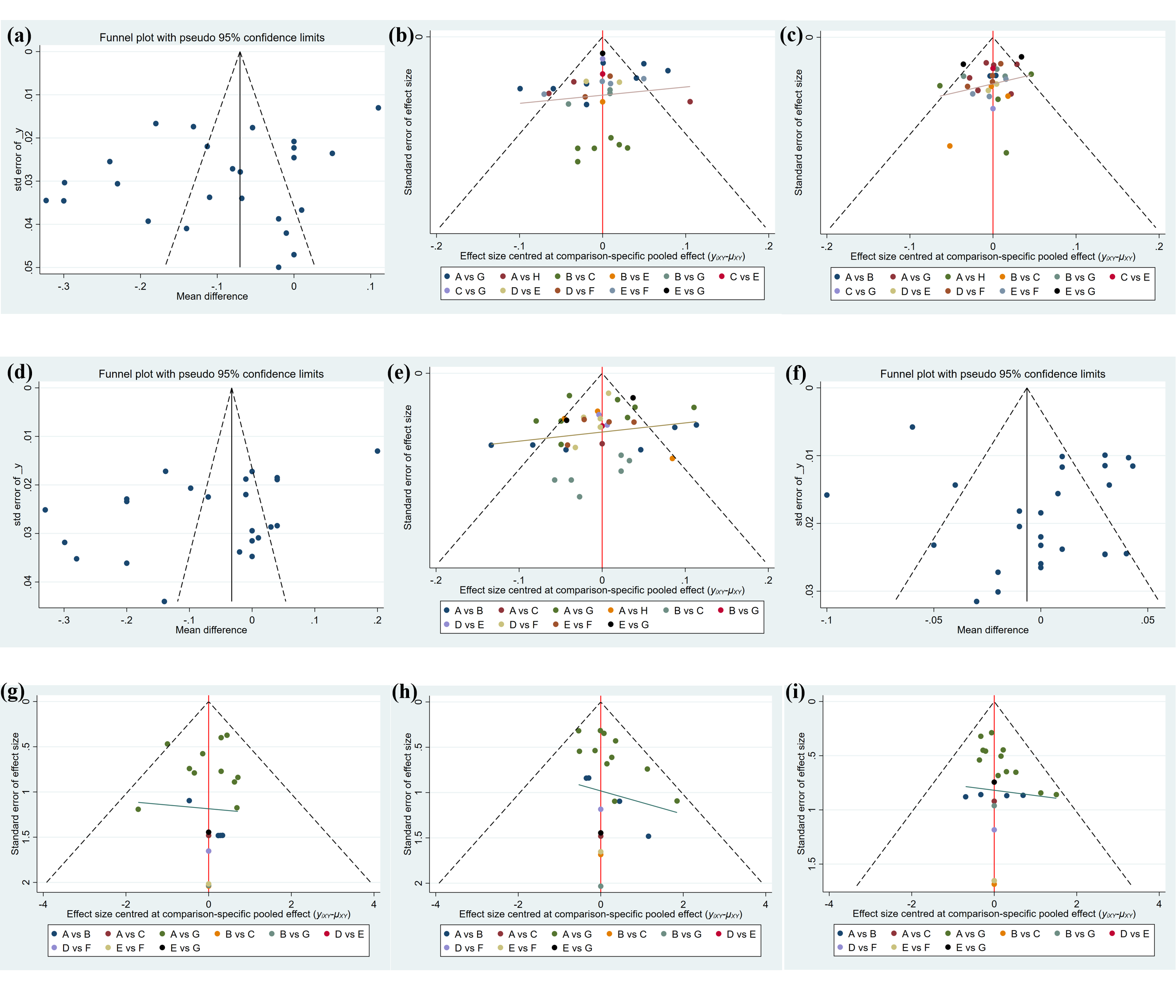
**

**Supplementary Figure 4.** Funnel plot of primary indicators

Note: A: standard monofocal IOLs group, B: bifocal IOLs group, C: AT LISAtri 839MP IOLs group, D: FineVision POD F IOLs group, E: AcrySof IQ PanOptix IOLs group, F: other new trifocal IOLs group, G: extended depth-of-focus(EDOF) IOLs group, H: enhanced monofocal IOLs group; (a): Funnel plot of UNVA, (b): Funnel plot of UIVA, (c): Funnel plot of UDVA, (d): Funnel plot of CNVA, (e): Funnel plot of CIVA, (f): Funnel plot of CDVA, (g): Funnel plot of distant spectacle independence, (h): Funnel plot of intermediate spectacle independence, (i): Funnel plot of near spectacle independence

**
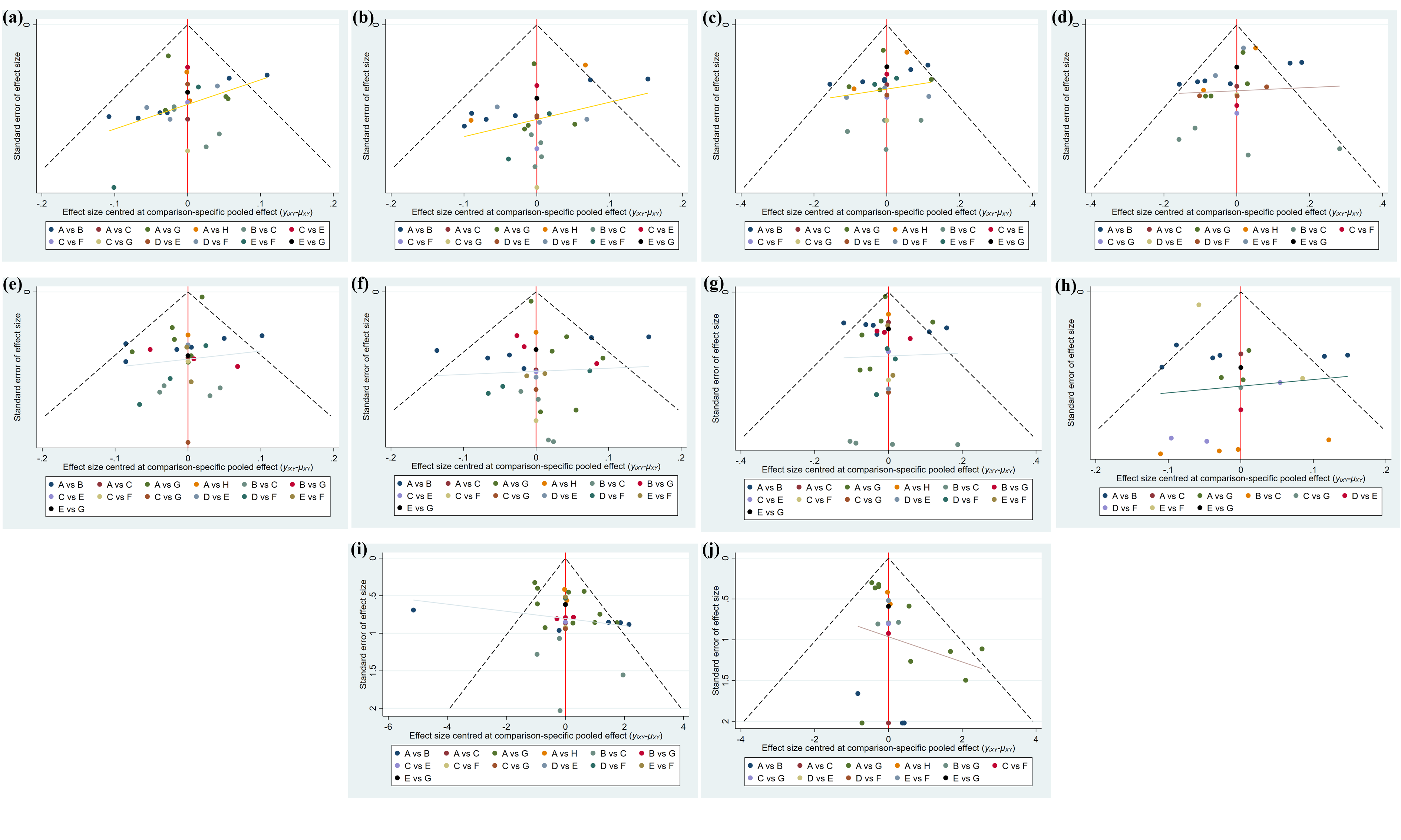
**

**Supplementary Figure 5.** Funnel plot of secondary indicators

Note:A: standard monofocal IOLs group, B: bifocal IOLs group, C: AT LISAtri 839MP IOLs group, D: FineVision POD F IOLs group, E: AcrySof IQ PanOptix IOLs group, F: other new trifocal IOLs group, G: extended depth-of-focus(EDOF) IOLs group, H: enhanced monofocal IOLs group; (a): Funnel plot of CS under photopic conditions at spatial frequencies of 3cpd, (b): Funnel plot of CS under photopic conditions at spatial frequencies of 6cpd, (c): Funnel plot of CS under photopic conditions at spatial frequencies of 12cpd, (d): Funnel plot of CS under photopic conditions at spatial frequencies of 18cpd, (e): Funnel plot of CS under mesopic conditions at spatial frequencies of 3cpd, (f): Funnel plot of CS under mesopic conditions at spatial frequencies of 6cpd, (g): Funnel plot of CS under mesopic conditions at spatial frequencies of 12cpd, (h): Funnel plot of CS under mesopic conditions at spatial frequencies of 18cpd, (i): Funnel plot of Halos, (j): Funnel plot of Glare

**
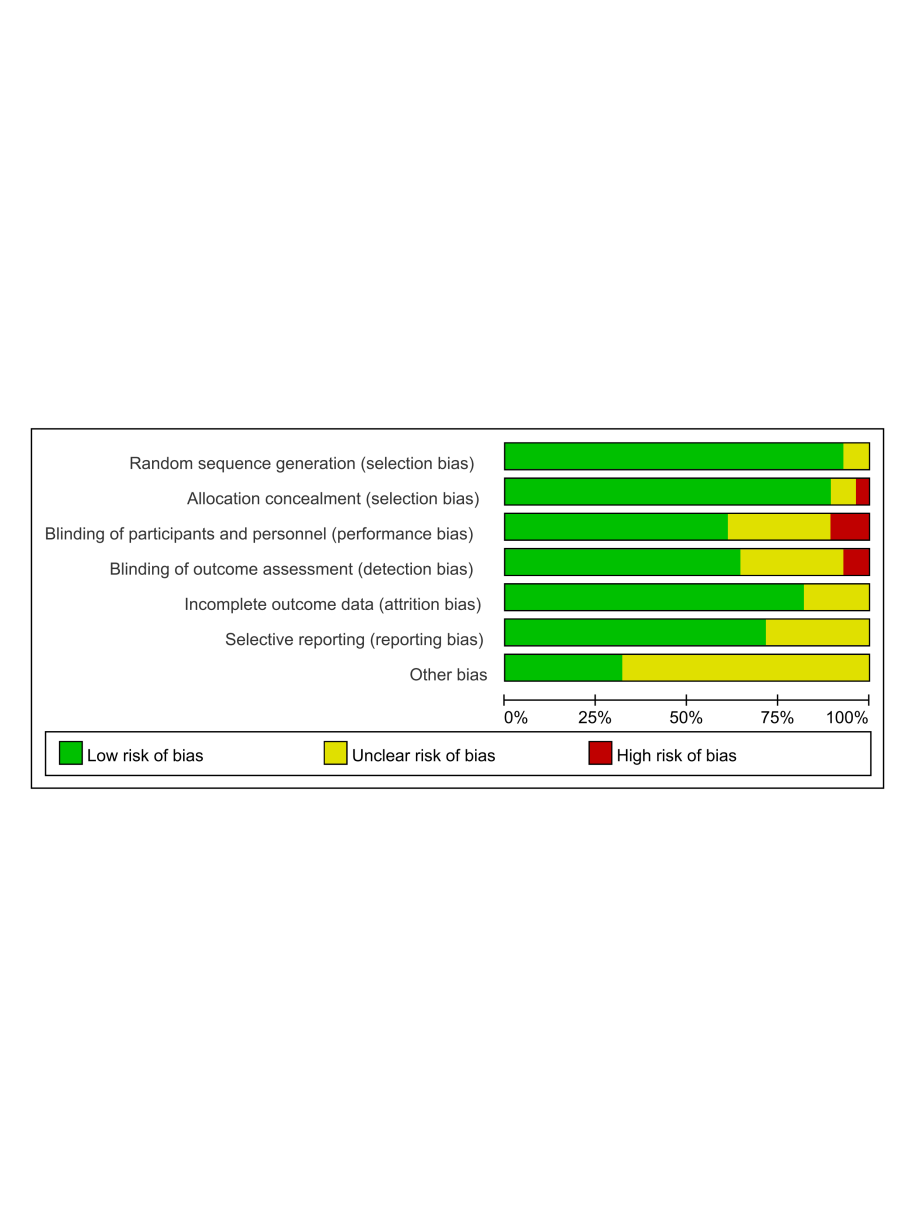
Supplementary Figure 6.** Risk of bias graph

**Supplementary table 1.** League tables for all outcome indicators

1. League tables of UNVA

| F | 0.02 (-0.08,0.11) | 0.03 (-0.05,0.11) | 0.10 (-0.02,0.23) | 0.13 (0.00,0.25) | 0.15 (0.02,0.28) | 0.35 (0.22,0.48) |
| --- | --- | --- | --- | --- | --- | --- |
| -0.02 (-0.11,0.08) | D | 0.02 (-0.08,0.11) | 0.09 (-0.05,0.22) | 0.11 (-0.02,0.24) | 0.14 (-0.00,0.28) | 0.34 (0.19,0.48) |
| -0.03 (-0.11,0.05) | -0.02 (-0.11,0.08) | E | 0.07 (-0.03,0.17) | 0.09 (-0.00,0.19) | 0.12 (0.02,0.22) | 0.32 (0.21,0.42) |
| -0.10 (-0.23,0.02) | -0.09 (-0.22,0.05) | -0.07 (-0.17,0.03) | C | 0.02 (-0.05,0.10) | 0.05 (-0.02,0.12) | 0.25 (0.16,0.33) |
| -0.13 (-0.25, -0.00) | -0.11 (-0.24,0.02) | -0.09 (-0.19,0.00) | -0.02 (-0.10,0.05) | G | 0.03 (-0.03,0.08) | 0.22 (0.18,0.27) |
| -0.15 (-0.28, -0.02) | -0.14 (-0.28,0.00) | -0.12 (-0.22, -0.02) | -0.05 (-0.12,0.02) | -0.03 (-0.08,0.03) | B | 0.20 (0.14,0.26) |
| -0.35 (-0.48, -0.22) | -0.34 (-0.48, -0.19) | -0.32 (-0.42, -0.21) | -0.25 (-0.33, -0.16) | -0.22 (-0.27, -0.18) | -0.20 (-0.26, -0.14) | A |

1. League tables of UIVA

| E | 0.00 (-0.06,0.07) | 0.01 (-0.04,0.06) | 0.02 (-0.05,0.08) | 0.02 (-0.04,0.09) | 0.07 (-0.03,0.16) | 0.08 (0.01,0.14) | 0.13 (0.06,0.21) |
| --- | --- | --- | --- | --- | --- | --- | --- |
| -0.00 (-0.07,0.06) | G | 0.01 (-0.08,0.09) | 0.01 (-0.08,0.11) | 0.02 (-0.04,0.08) | 0.06 (-0.01,0.14) | 0.07 (0.03,0.12) | 0.13 (0.09,0.17) |
| -0.01 (-0.06,0.04) | -0.01 (-0.09,0.08) | F | 0.01 (-0.06,0.08) | 0.01 (-0.07,0.10) | 0.06 (-0.05,0.17) | 0.07 (-0.02,0.15) | 0.12 (0.03,0.22) |
| -0.02 (-0.08,0.05) | -0.01 (-0.11,0.08) | -0.01 (-0.08,0.06) | D | 0.01 (-0.09,0.11) | 0.05 (-0.07,0.17) | 0.06 (-0.03,0.16) | 0.12 (0.02,0.22) |
| -0.02 (-0.09,0.04) | -0.02 (-0.08,0.04) | -0.01 (-0.10,0.07) | -0.01 (-0.11,0.09) | C | 0.04 (-0.05,0.13) | 0.05 (0.00,0.10) | 0.11 (0.04,0.18) |
| -0.07 (-0.16,0.03) | -0.06 (-0.14,0.01) | -0.06 (-0.17,0.05) | -0.05 (-0.17,0.07) | -0.04 (-0.13,0.05) | H | 0.01 (-0.08,0.10) | 0.07 (0.00,0.13) |
| -0.08 (-0.14, -0.01) | -0.07 (-0.12, -0.03) | -0.07 (-0.15,0.02) | -0.06 (-0.16,0.03) | -0.05 (-0.10, -0.00) | -0.01 (-0.10,0.08) | B | 0.06 (-0.00,0.12) |
| -0.13 (-0.21, -0.06) | -0.13 (-0.17, -0.09) | -0.12 (-0.22, -0.03) | -0.12 (-0.22, -0.02) | -0.11 (-0.18, -0.04) | -0.07 (-0.13, -0.00) | -0.06 (-0.12,0.00) | A |

1. League tables of UDVA

| A | 0.02 (-0.02,0.05) | 0.02 (-0.01,0.04) | 0.03 (0.00,0.05) | 0.04 (-0.00,0.08) | 0.08 (0.02,0.13) | 0.08 (0.05,0.12) | 0.09 (0.04,0.14) |
| --- | --- | --- | --- | --- | --- | --- | --- |
| -0.02 (-0.05,0.02) | H | 0.00 (-0.04,0.05) | 0.01 (-0.03,0.05) | 0.02 (-0.03,0.08) | 0.06 (-0.01,0.13) | 0.07 (0.02,0.12) | 0.08 (0.01,0.14) |
| -0.02 (-0.04,0.01) | -0.00 (-0.05,0.04) | B | 0.01 (-0.02,0.03) | 0.02 (-0.01,0.06) | 0.06 (0.00,0.11) | 0.07 (0.03,0.10) | 0.07 (0.02,0.13) |
| -0.03 (-0.05, -0.00) | -0.01 (-0.05,0.03) | -0.01 (-0.03,0.02) | G | 0.02 (-0.02,0.05) | 0.05 (-0.00,0.10) | 0.06 (0.03,0.09) | 0.07 (0.02,0.12) |
| -0.04 (-0.08,0.00) | -0.02 (-0.08,0.03) | -0.02 (-0.06,0.01) | -0.02 (-0.05,0.02) | C | 0.04 (-0.02,0.09) | 0.04 (0.00,0.08) | 0.05 (-0.00,0.11) |
| -0.08 (-0.13, -0.02) | -0.06 (-0.13,0.01) | -0.06 (-0.11, -0.00) | -0.05 (-0.10,0.00) | -0.04 (-0.09,0.02) | D | 0.01 (-0.03,0.05) | 0.02 (-0.01,0.04) |
| -0.08 (-0.12, -0.05) | -0.07 (-0.12, -0.02) | -0.07 (-0.10, -0.03) | -0.06 (-0.09, -0.03) | -0.04 (-0.08, -0.00) | -0.01 (-0.05,0.03) | E | 0.01 (-0.03,0.05) |
| -0.09 (-0.14, -0.04) | -0.08 (-0.14, -0.01) | -0.07 (-0.13, -0.02) | -0.07 (-0.12, -0.02) | -0.05 (-0.11,0.00) | -0.02 (-0.04,0.01) | -0.01 (-0.05,0.03) | F |

1. League tables of CNVA

| E | 0.00 (-0.08,0.08) | 0.02 (-0.05,0.09) | 0.06 (-0.09,0.22) | 0.07 (-0.05,0.19) | 0.12 (0.03,0.21) | 0.32 (0.22,0.42) |
| --- | --- | --- | --- | --- | --- | --- |
| -0.00 (-0.08, 0.08) | D | 0.02 (-0.05,0.08) | 0.06 (-0.11,0.24) | 0.07 (-0.08,0.21) | 0.12 (0.00,0.24) | 0.32 (0.19,0.44) |
| -0.02 (-0.09, 0.05) | -0.02 (-0.08, 0.05) | F | 0.04 (-0.13,0.22) | 0.05 (-0.09,0.19) | 0.10 (-0.01,0.22) | 0.30 (0.18,0.42) |
| -0.06 (-0.22,0.09) | -0.06 (-0.24, 0.11) | -0.04 (-0.22,0.13) | C | 0.00 (-0.09,0.10) | 0.06 (-0.07,0.18) | 0.25 (0.13,0.38) |
| -0.07 (-0.19,0.05) | -0.07 (-0.21,0.08) | -0.05 (-0.19,0.09) | -0.00 (-0.10,0.09) | B | 0.05 (-0.03,0.13) | 0.25 (0.17,0.33) |
| -0.12 (-0.21, -0.03) | -0.12 (-0.24, -0.00) | -0.10 (-0.22,0.01) | -0.06 (-0.18,0.07) | -0.05 (-0.13,0.03) | G | 0.20 (0.15,0.24) |
| -0.32 (-0.42, -0.22) | -0.32 (-0.44, -0.19) | -0.30 (-0.42, -0.18) | -0.25 (-0.38, -0.13) | -0.25 (-0.33, -0.17) | -0.20 (-0.24, -0.15) | A |

1. League tables of CIVA

| E | 0.01 (-0.04,0.07) | 0.02 (-0.04,0.08) | 0.07 (-0.03,0.18) | 0.07 (-0.00,0.15) | 0.13 (0.03,0.22) | 0.14 (0.03,0.24) | 0.24 (0.15,0.32) |
| --- | --- | --- | --- | --- | --- | --- | --- |
| -0.01 (-0.07,0.04) | F | 0.01 (-0.04,0.06) | 0.06 (-0.06,0.18) | 0.06 (-0.03,0.16) | 0.11 (0.01,0.22) | 0.12 (0.00,0.25) | 0.23 (0.13,0.33) |
| -0.02 (-0.08,0.04) | -0.01 (-0.06,0.04) | D | 0.05 (-0.07,0.17) | 0.05 (-0.05,0.15) | 0.10 (-0.01,0.22) | 0.11 (-0.01,0.24) | 0.22 (0.11,0.32) |
| -0.07 (-0.18,0.03) | -0.06 (-0.18,0.06) | -0.05 (-0.17,0.07) | C | 0.00 (-0.07,0.07) | 0.05 (0.00,0.11) | 0.07 (-0.03,0.16) | 0.17 (0.10,0.23) |
| -0.07 (-0.15,0.00) | -0.06 (-0.16,0.03) | -0.05 (-0.15,0.05) | -0.00 (-0.07,0.07) | G | 0.05 (-0.00,0.11) | 0.06 (-0.01,0.14) | 0.17 (0.13,0.20) |
| -0.13 (-0.22,-0.03) | -0.11 (-0.22,-0.01) | -0.10 (-0.22,0.01) | -0.05 (-0.11,-0.00) | -0.05 (-0.11,0.00) | B | 0.01 (-0.07,0.09) | 0.11 (0.07,0.16) |
| -0.14 (-0.24,-0.03) | -0.12 (-0.25,-0.00) | -0.11 (-0.24,0.01) | -0.07 (-0.16,0.03) | -0.06 (-0.14,0.01) | -0.01 (-0.09,0.07) | H | 0.10 (0.03,0.17) |
| -0.24 (-0.32,-0.15) | -0.23 (-0.33,-0.13) | -0.22 (-0.32,-0.11) | -0.17 (-0.23,-0.10) | -0.17 (-0.20,-0.13) | -0.11 (-0.16,-0.07) | -0.10 (-0.17,-0.03) | A |

1. League tables of CDVA

| H | 0.01 (-0.04,0.06) | 0.01 (-0.01,0.03) | 0.04 (0.00,0.07) | 0.04 (0.01,0.07) | 0.10 (0.05,0.15) | 0.11 (0.08,0.15) | 0.11 (0.07,0.16) |
| --- | --- | --- | --- | --- | --- | --- | --- |
| -0.01 (-0.06,0.04) | C | 0.00 (-0.04,0.05) | 0.03 (-0.01,0.07) | 0.03 (-0.02,0.08) | 0.09 (0.03,0.15) | 0.10 (0.05,0.16) | 0.10 (0.04,0.17) |
| -0.01 (-0.03,0.01) | -0.00 (-0.05,0.04) | A | 0.03 (0.00,0.05) | 0.03 (0.01,0.04) | 0.09 (0.05,0.13) | 0.10 (0.07,0.13) | 0.10 (0.06,0.14) |
| -0.04 (-0.07,-0.00) | -0.03 (-0.07,0.01) | -0.03 (-0.05,-0.00) | B | 0.00 (-0.02,0.03) | 0.06 (0.02,0.11) | 0.08 (0.04,0.11) | 0.08 (0.03,0.12) |
| -0.04 (-0.07,-0.01) | -0.03 (-0.08,0.02) | -0.03 (-0.04,-0.01) | -0.00 (-0.03,0.02) | G | 0.06 (0.02,0.10) | 0.07 (0.05,0.10) | 0.07 (0.04,0.11) |
| -0.10 (-0.15,-0.05) | -0.09 (-0.15,-0.03) | -0.09 (-0.13,-0.05) | -0.06 (-0.11,-0.02) | -0.06 (-0.10,-0.02) | D | 0.01 (-0.02,0.04) | 0.01 (-0.01,0.03) |
| -0.11 (-0.15,-0.08) | -0.10 (-0.16,-0.05) | -0.10 (-0.13,-0.07) | -0.08 (-0.11,-0.04) | -0.07 (-0.10,-0.05) | -0.01 (-0.04,0.02) | E | -0.00 (-0.03,0.03) |
| -0.11 (-0.16,-0.07) | -0.10 (-0.17,-0.04) | -0.10 (-0.14,-0.06) | -0.08 (-0.12,-0.03) | -0.07 (-0.11,-0.04) | -0.01 (-0.03,0.01) | 0.00 (-0.03,0.03) | F |

(g) League tables of distant spectacle independence

| C | 0.06 (0.00,0.77) | 0.02 (0.00,4.24) | 0.02 (0.00,0.76) | 0.02 (0.00,0.18) | 0.01 (0.00,0.12) | 0.01 (0.00,0.84) |
| --- | --- | --- | --- | --- | --- | --- |
| 16.17 (1.30,201.76) | B | 0.28 (0.00,45.44) | 0.28 (0.01,6.60) | 0.25 (0.07,0.93) | 0.17 (0.05,0.60) | 0.09 (0.00,8.63) |
| 58.31 (0.24,14435.45) | 3.61 (0.02,591.15) | F | 1.00 (0.02,54.30) | 0.92 (0.01,126.96) | 0.62 (0.00,87.08) | 0.32 (0.01,8.67) |
| 58.31 (1.31,2600.18) | 3.61 (0.15,85.82) | 1.00 (0.02,54.30) | E | 0.92 (0.05,16.51) | 0.62 (0.03,11.46) | 0.32 (0.01,8.67) |
| 63.62 (5.42,746.53) | 3.93 (1.07,14.43) | 1.09 (0.01,151.09) | 1.09 (0.06,19.64) | G | 0.67 (0.45,1.02) | 0.35 (0.00,28.12) |
| 94.38 (8.32,1070.48) | 5.84 (1.68,20.28) | 1.62 (0.01,228.09) | 1.62 (0.09,30.02) | 1.48 (0.98,2.25) | A | 0.52 (0.01,42.54) |
| 180.87 (1.19,27549.17) | 11.19 (0.12,1080.02) | 3.10 (0.12,83.45) | 3.10 (0.12,83.45) | 2.84 (0.04,227.29) | 1.92 (0.02,156.25) | D |

(h) League tables of intermediate spectacle independence

| C | 0.42 (0.01,15.59) | 0.39 (0.04,3.62) | 0.35 (0.04,3.33) | 0.14 (0.00,17.40) | 0.14 (0.00,17.40) | 0.09 (0.01,0.79) |
| --- | --- | --- | --- | --- | --- | --- |
| 2.36 (0.06,86.97) | E | 0.92 (0.05,15.56) | 0.84 (0.04,16.61) | 0.32 (0.01,8.24) | 0.32 (0.01,8.24) | 0.20 (0.01,3.50) |
| 2.58 (0.28,24.03) | 1.09 (0.06,18.51) | G | 0.91 (0.35,2.37) | 0.35 (0.00,26.00) | 0.35 (0.00,26.00) | 0.22 (0.17,0.29) |
| 2.82 (0.30,26.52) | 1.20 (0.06,23.72) | 1.10 (0.42,2.85) | B | 0.39 (0.00,31.63) | 0.39 (0.00,31.63) | 0.24 (0.10,0.61) |
| 7.33 (0.06,934.01) | 3.10 (0.12,79.23) | 2.84 (0.04,210.22) | 2.60 (0.03,213.06) | D | 1.00 (0.10,10.18) | 0.63 (0.01,47.01) |
| 7.33 (0.06,934.01) | 3.10 (0.12,79.23) | 2.84 (0.04,210.22) | 2.60 (0.03,213.06) | 1.00 (0.10,10.18) | F | 0.63 (0.01,47.01) |
| 11.63 (1.27,106.63) | 4.92 (0.29,84.70) | 4.51 (3.41,5.97) | 4.12 (1.65,10.31) | 1.59 (0.02,118.41) | 1.59 (0.02,118.41) | A |

(i) League tables of near spectacle independence

| E | 0.80 (0.09,7.02) | 0.34 (0.06,1.77) | 0.32 (0.01,8.24) | 0.32 (0.01,8.24) | 0.15 (0.03,0.64) | 0.03 (0.01,0.11) |
| --- | --- | --- | --- | --- | --- | --- |
| 1.25 (0.14,11.01) | C | 0.42 (0.08,2.29) | 0.40 (0.01,19.98) | 0.40 (0.01,19.98) | 0.19 (0.04,0.93) | 0.03 (0.01,0.16) |
| 2.96 (0.56,15.55) | 2.37 (0.44,12.79) | B | 0.95 (0.03,36.38) | 0.95 (0.03,36.38) | 0.44 (0.20,0.97) | 0.08 (0.04,0.16) |
| 3.10 (0.12,79.23) | 2.48 (0.05,122.59) | 1.05 (0.03,39.89) | F | 1.00 (0.10,10.18) | 0.46 (0.01,16.11) | 0.08 (0.00,2.79) |
| 3.10 (0.12,79.23) | 2.48 (0.05,122.59) | 1.05 (0.03,39.89) | 1.00 (0.10,10.18) | D | 0.46 (0.01,16.11) | 0.08 (0.00,2.79) |
| 6.72 (1.56,28.87) | 5.37 (1.07,26.92) | 2.27 (1.03,5.01) | 2.17 (0.06,75.68) | 2.17 (0.06,75.68) | G | 0.17 (0.13,0.22) |
| 39.27 (8.93,172.78) | 31.36 (6.38,154.09) | 13.26 (6.20,28.36) | 12.66 (0.36,446.53) | 12.66 (0.36,446.53) | 5.84 (4.48,7.62) | A |

(j) League tables of under photopic conditions at spatial frequencies of 3cpd

| A | -0.00 (-0.09,0.08) | -0.07 (-0.12,-0.01) | -0.08 (-0.19,0.03) | -0.09 (-0.21,0.04) | -0.09 (-0.18,-0.00) | -0.13 (-0.20,-0.06) | -0.14 (-0.19,-0.09) |
| --- | --- | --- | --- | --- | --- | --- | --- |
| 0.00 (-0.08,0.09) | H | -0.07 (-0.17,0.03) | -0.08 (-0.22,0.06) | -0.08 (-0.23,0.06) | -0.09 (-0.21,0.03) | -0.13 (-0.23,-0.02) | -0.13 (-0.23,-0.04) |
| 0.07 (0.01,0.12) | 0.07 (-0.03,0.17) | G | -0.01 (-0.12,0.10) | -0.02 (-0.14,0.11) | -0.02 (-0.11,0.07) | -0.06 (-0.14,0.02) | -0.07 (-0.14,0.00) |
| 0.08 (-0.03,0.19) | 0.08 (-0.06,0.22) | 0.01 (-0.10,0.12) | F | -0.00 (-0.08,0.08) | -0.01 (-0.09,0.08) | -0.04 (-0.14,0.05) | -0.05 (-0.16,0.06) |
| 0.09 (-0.04,0.21) | 0.08 (-0.06,0.23) | 0.02 (-0.11,0.14) | 0.00 (-0.08,0.08) | D | -0.01 (-0.10,0.09) | -0.04 (-0.15,0.07) | -0.05 (-0.17,0.07) |
| 0.09 (0.00,0.18) | 0.09 (-0.03,0.21) | 0.02 (-0.07,0.11) | 0.01 (-0.08,0.09) | 0.01 (-0.09,0.10) | E | -0.04 (-0.11,0.04) | -0.04 (-0.14,0.05) |
| 0.13 (0.06,0.20) | 0.13 (0.02,0.23) | 0.06 (-0.02,0.14) | 0.04 (-0.05,0.14) | 0.04 (-0.07,0.15) | 0.04 (-0.04,0.11) | C | -0.01 (-0.07,0.06) |
| 0.14 (0.09,0.19) | 0.13 (0.04,0.23) | 0.07 (-0.00,0.14) | 0.05 (-0.06,0.16) | 0.05 (-0.07,0.17) | 0.04 (-0.05,0.14) | 0.01 (-0.06,0.07) | B |

(k) League tables of under photopic conditions at spatial frequencies of 6cpd

| A | -0.02 (-0.21,0.17) | -0.02 (-0.15,0.10) | -0.04 (-0.18,0.10) | -0.04 (-0.21,0.12) | -0.16 (-0.26,-0.06) | -0.19 (-0.28,-0.10) | -0.21 (-0.28,-0.14) |
| --- | --- | --- | --- | --- | --- | --- | --- |
| 0.02 (-0.17,0.21) | D | -0.01 (-0.23,0.22) | -0.02 (-0.17,0.13) | -0.03 (-0.14,0.09) | -0.14 (-0.31,0.03) | -0.17 (-0.36,0.02) | -0.19 (-0.38,-0.00) |
| 0.02 (-0.10,0.15) | 0.01 (-0.22,0.23) | H | -0.02 (-0.20,0.17) | -0.02 (-0.23,0.19) | -0.13 (-0.29,0.03) | -0.17 (-0.32,-0.01) | -0.19 (-0.33,-0.04) |
| 0.04 (-0.10,0.18) | 0.02 (-0.13,0.17) | 0.02 (-0.17,0.20) | E | -0.00 (-0.13,0.12) | -0.12 (-0.24,0.01) | -0.15 (-0.28,-0.02) | -0.17 (-0.31,-0.03) |
| 0.04 (-0.12,0.21) | 0.03 (-0.09,0.14) | 0.02 (-0.19,0.23) | 0.00 (-0.12,0.13) | F | -0.11 (-0.26,0.04) | -0.15 (-0.32,0.02) | -0.17 (-0.34,-0.00) |
| 0.16 (0.06,0.26) | 0.14 (-0.03,0.31) | 0.13 (-0.03,0.29) | 0.12 (-0.01,0.24) | 0.11 (-0.04,0.26) | C | -0.03 (-0.15,0.08) | -0.05 (-0.15,0.04) |
| 0.19 (0.10,0.28) | 0.17 (-0.02,0.36) | 0.17 (0.01,0.32) | 0.15 (0.02,0.28) | 0.15 (-0.02,0.32) | 0.03 (-0.08,0.15) | G | -0.02 (-0.13,0.08) |
| 0.21 (0.14,0.28) | 0.19 (0.00,0.38) | 0.19 (0.04,0.33) | 0.17 (0.03,0.31) | 0.17 (0.00,0.34) | 0.05 (-0.04,0.15) | 0.02 (-0.08,0.13) | B |

(l) League tables of under photopic conditions at spatial frequencies of 12cpd

| A | -0.00 (-0.12,0.11) | -0.03 (-0.21,0.16) | -0.07 (-0.23,0.08) | -0.14 (-0.27,-0.01) | -0.16 (-0.24,-0.09) | -0.23 (-0.30,-0.16) | -0.23 (-0.34,-0.13) |
| --- | --- | --- | --- | --- | --- | --- | --- |
| 0.00 (-0.11,0.12) | H | -0.02 (-0.24,0.19) | -0.07 (-0.26,0.12) | -0.14 (-0.31,0.03) | -0.16 (-0.30,-0.02) | -0.23 (-0.36,-0.09) | -0.23 (-0.38,-0.08) |
| 0.03 (-0.16,0.21) | 0.02 (-0.19,0.24) | D | -0.04 (-0.16,0.07) | -0.12 (-0.26,0.03) | -0.14 (-0.32,0.04) | -0.20 (-0.39,-0.02) | -0.21 (-0.38,-0.04) |
| 0.07 (-0.08,0.23) | 0.07 (-0.12,0.26) | 0.04 (-0.07,0.16) | F | -0.07 (-0.18,0.04) | -0.09 (-0.25,0.06) | -0.16 (-0.32,0.00) | -0.16 (-0.30,-0.03) |
| 0.14 (0.01,0.27) | 0.14 (-0.03,0.31) | 0.12 (-0.03,0.26) | 0.07 (-0.04,0.18) | E | -0.02 (-0.14,0.10) | -0.09 (-0.22,0.05) | -0.09 (-0.21,0.02) |
| 0.16 (0.09,0.24) | 0.16 (0.02,0.30) | 0.14 (-0.04,0.32) | 0.09 (-0.06,0.25) | 0.02 (-0.10,0.14) | G | -0.07 (-0.16,0.03) | -0.07 (-0.18,0.04) |
| 0.23 (0.16,0.30) | 0.23 (0.09,0.36) | 0.20 (0.02,0.39) | 0.16 (-0.00,0.32) | 0.09 (-0.05,0.22) | 0.07 (-0.03,0.16) | B | -0.00 (-0.11,0.10) |
| 0.23 (0.13,0.34) | 0.23 (0.08,0.38) | 0.21 (0.04,0.38) | 0.16 (0.03,0.30) | 0.09 (-0.02,0.21) | 0.07 (-0.04,0.18) | 0.00 (-0.10,0.11) | C |

(m) League tables of under photopic conditions at spatial frequencies of 18cpd

| A | -0.06 (-0.30,0.18) | -0.06 (-0.21,0.09) | -0.15 (-0.36,0.05) | -0.22 (-0.32,-0.11) | -0.22 (-0.41,-0.02) | -0.30 (-0.38,-0.21) | -0.34 (-0.46,-0.21) |
| --- | --- | --- | --- | --- | --- | --- | --- |
| 0.06 (-0.18,0.30) | D | -0.01 (-0.29,0.28) | -0.10 (-0.24,0.04) | -0.16 (-0.39,0.07) | -0.16 (-0.33,0.01) | -0.24 (-0.49,0.01) | -0.28 (-0.51,-0.04) |
| 0.06 (-0.09,0.21) | 0.01 (-0.28,0.29) | H | -0.09 (-0.34,0.16) | -0.16 (-0.34,0.03) | -0.15 (-0.40,0.09) | -0.23 (-0.41,-0.06) | -0.27 (-0.47,-0.08) |
| 0.15 (-0.05,0.36) | 0.10 (-0.04,0.24) | 0.09 (-0.16,0.34) | F | -0.06 (-0.26,0.13) | -0.06 (-0.19,0.07) | -0.14 (-0.35,0.07) | -0.18 (-0.38,0.02) |
| 0.22 (0.11,0.32) | 0.16 (-0.07,0.39) | 0.16 (-0.03,0.34) | 0.06 (-0.13,0.26) | G | 0.00 (-0.18,0.18) | -0.08 (-0.21,0.05) | -0.12 (-0.26,0.03) |
| 0.22 (0.02,0.41) | 0.16 (-0.01,0.33) | 0.15 (-0.09,0.40) | 0.06 (-0.07,0.19) | -0.00 (-0.18,0.18) | E | -0.08 (-0.28,0.12) | -0.12 (-0.32,0.08) |
| 0.30 (0.21,0.38) | 0.24 (-0.01,0.49) | 0.23 (0.06,0.41) | 0.14 (-0.07,0.35) | 0.08 (-0.05,0.21) | 0.08 (-0.12,0.28) | B | -0.04 (-0.17,0.09) |
| 0.34 (0.21,0.46) | 0.28 (0.04,0.51) | 0.27 (0.08,0.47) | 0.18 (-0.02,0.38) | 0.12 (-0.03,0.26) | 0.12 (-0.08,0.32) | 0.04 (-0.09,0.17) | C |

(n) League tables of under mesopic conditions at spatial frequencies of 3cpd

| H | -0.05 (-0.14,0.05) | -0.12 (-0.22,-0.02) | -0.13 (-0.28,0.02) | -0.17 (-0.31,-0.03) | -0.17 (-0.30,-0.04) | -0.18 (-0.30,-0.07) | -0.19 (-0.29,-0.08) |
| --- | --- | --- | --- | --- | --- | --- | --- |
| 0.05 (-0.05,0.14) | A | -0.07 (-0.11,-0.04) | -0.08 (-0.20,0.03) | -0.12 (-0.22,-0.02) | -0.12 (-0.21,-0.04) | -0.13 (-0.20,-0.07) | -0.14 (-0.17,-0.10) |
| 0.12 (0.02,0.22) | 0.07 (0.04,0.11) | G | -0.01 (-0.13,0.11) | -0.04 (-0.15,0.06) | -0.05 (-0.13,0.03) | -0.06 (-0.13,0.01) | -0.06 (-0.11,-0.02) |
| 0.13 (-0.02,0.28) | 0.08 (-0.03,0.20) | 0.01 (-0.11,0.13) | D | -0.04 (-0.11,0.04) | -0.04 (-0.13,0.05) | -0.05 (-0.16,0.06) | -0.06 (-0.17,0.06) |
| 0.17 (0.03,0.31) | 0.12 (0.02,0.22) | 0.04 (-0.06,0.15) | 0.04 (-0.04,0.11) | F | -0.01 (-0.08,0.07) | -0.02 (-0.10,0.07) | -0.02 (-0.12,0.08) |
| 0.17 (0.04,0.30) | 0.12 (0.04,0.21) | 0.05 (-0.03,0.13) | 0.04 (-0.05,0.13) | 0.01 (-0.07,0.08) | E | -0.01 (-0.08,0.07) | -0.01 (-0.10,0.07) |
| 0.18 (0.07,0.30) | 0.13 (0.07,0.20) | 0.06 (-0.01,0.13) | 0.05 (-0.06,0.16) | 0.02 (-0.07,0.10) | 0.01 (-0.07,0.08) | C | -0.00 (-0.07,0.06) |
| 0.19 (0.08,0.29) | 0.14 (0.10,0.17) | 0.06 (0.02,0.11) | 0.06 (-0.06,0.17) | 0.02 (-0.08,0.12) | 0.01 (-0.07,0.10) | 0.00 (-0.06,0.07) | B |

(o) League tables of under mesopic conditions at spatial frequencies of 6cpd

| H | -0.07 (-0.19,0.05) | -0.22 (-0.42,-0.01) | -0.24 (-0.37,-0.11) | -0.26 (-0.45,-0.07) | -0.29 (-0.42,-0.16) | -0.29 (-0.46,-0.13) | -0.31 (-0.45,-0.16) |
| --- | --- | --- | --- | --- | --- | --- | --- |
| 0.07 (-0.05,0.19) | A | -0.15 (-0.32,0.02) | -0.18 (-0.22,-0.13) | -0.20 (-0.34,-0.05) | -0.22 (-0.27,-0.18) | -0.23 (-0.34,-0.11) | -0.24 (-0.32,-0.15) |
| 0.22 (0.01,0.42) | 0.15 (-0.02,0.32) | D | -0.03 (-0.19,0.14) | -0.05 (-0.15,0.06) | -0.07 (-0.24,0.09) | -0.08 (-0.21,0.06) | -0.09 (-0.25,0.07) |
| 0.24 (0.11,0.37) | 0.18 (0.13,0.22) | 0.03 (-0.14,0.19) | G | -0.02 (-0.16,0.12) | -0.05 (-0.10,0.01) | -0.05 (-0.16,0.06) | -0.06 (-0.15,0.02) |
| 0.26 (0.07,0.45) | 0.20 (0.05,0.34) | 0.05 (-0.06,0.15) | 0.02 (-0.12,0.16) | F | -0.03 (-0.17,0.12) | -0.03 (-0.14,0.08) | -0.04 (-0.18,0.10) |
| 0.29 (0.16,0.42) | 0.22 (0.18,0.27) | 0.07 (-0.09,0.24) | 0.05 (-0.01,0.10) | 0.03 (-0.12,0.17) | B | -0.00 (-0.12,0.11) | -0.01 (-0.09,0.07) |
| 0.29 (0.13,0.46) | 0.23 (0.11,0.34) | 0.08 (-0.06,0.21) | 0.05 (-0.06,0.16) | 0.03 (-0.08,0.14) | 0.00 (-0.11,0.12) | E | -0.01 (-0.12,0.10) |
| 0.31 (0.16,0.45) | 0.24 (0.15,0.32) | 0.09 (-0.07,0.25) | 0.06 (-0.02,0.15) | 0.04 (-0.10,0.18) | 0.01 (-0.07,0.09) | 0.01 (-0.10,0.12) | C |

(p) League tables of under mesopic conditions at spatial frequencies of 12cpd

| A | 0.00 (-0.17,0.17) | -0.07 (-0.29,0.16) | -0.08 (-0.26,0.11) | -0.14 (-0.29,0.01) | -0.19 (-0.25,-0.12) | -0.27 (-0.34,-0.21) | -0.32 (-0.44,-0.20) |
| --- | --- | --- | --- | --- | --- | --- | --- |
| -0.00 (-0.17,0.17) | H | -0.07 (-0.35,0.21) | -0.08 (-0.33,0.17) | -0.14 (-0.37,0.09) | -0.19 (-0.37,-0.01) | -0.28 (-0.46,-0.10) | -0.32 (-0.53,-0.11) |
| 0.07 (-0.16,0.29) | 0.07 (-0.21,0.35) | D | -0.01 (-0.15,0.14) | -0.07 (-0.25,0.11) | -0.12 (-0.34,0.11) | -0.21 (-0.43,0.02) | -0.25 (-0.46,-0.03) |
| 0.08 (-0.11,0.26) | 0.08 (-0.17,0.33) | 0.01 (-0.14,0.15) | F | -0.06 (-0.20,0.07) | -0.11 (-0.29,0.07) | -0.20 (-0.39,-0.01) | -0.24 (-0.42,-0.07) |
| 0.14 (-0.01,0.29) | 0.14 (-0.09,0.37) | 0.07 (-0.11,0.25) | 0.06 (-0.07,0.20) | E | -0.05 (-0.19,0.10) | -0.14 (-0.29,0.02) | -0.18 (-0.33,-0.03) |
| 0.19 (0.12,0.25) | 0.19 (0.01,0.37) | 0.12 (-0.11,0.34) | 0.11 (-0.07,0.29) | 0.05 (-0.10,0.19) | G | -0.09 (-0.16,-0.01) | -0.13 (-0.25,-0.01) |
| 0.27 (0.21,0.34) | 0.28 (0.10,0.46) | 0.21 (-0.02,0.43) | 0.20 (0.01,0.39) | 0.14 (-0.02,0.29) | 0.09 (0.01,0.16) | B | -0.04 (-0.17,0.08) |
| 0.32 (0.20,0.44) | 0.32 (0.11,0.53) | 0.25 (0.03,0.46) | 0.24 (0.07,0.42) | 0.18 (0.03,0.33) | 0.13 (0.01,0.25) | 0.04 (-0.08,0.17) | C |

(q) League tables of under mesopic conditions at spatial frequencies of 18cpd

| D | -0.10 (-0.24,0.04) | -0.16 (-0.45,0.12) | -0.21 (-0.38,-0.04) | -0.28 (-0.54,-0.02) | -0.42 (-0.71,-0.13) | -0.50 (-0.79,-0.21) |
| --- | --- | --- | --- | --- | --- | --- |
| 0.10 (-0.04,0.24) | F | -0.07 (-0.33,0.20) | -0.11 (-0.24,0.02) | -0.18 (-0.42,0.06) | -0.32 (-0.59,-0.06) | -0.40 (-0.67,-0.13) |
| 0.16 (-0.12,0.45) | 0.07 (-0.20,0.33) | A | -0.04 (-0.27,0.18) | -0.12 (-0.22,-0.01) | -0.26 (-0.33,-0.19) | -0.34 (-0.45,-0.23) |
| 0.21 (0.04,0.38) | 0.11 (-0.02,0.24) | 0.04 (-0.18,0.27) | E | -0.07 (-0.27,0.13) | -0.21 (-0.45,0.02) | -0.29 (-0.53,-0.05) |
| 0.28 (0.02,0.54) | 0.18 (-0.06,0.42) | 0.12 (0.01,0.22) | 0.07 (-0.13,0.27) | G | -0.14 (-0.27,-0.02) | -0.22 (-0.35,-0.08) |
| 0.42 (0.13,0.71) | 0.32 (0.06,0.59) | 0.26 (0.19,0.33) | 0.21 (-0.02,0.45) | 0.14 (0.02,0.27) | B | -0.08 (-0.18,0.03) |
| 0.50 (0.21,0.79) | 0.40 (0.13,0.67) | 0.34 (0.23,0.45) | 0.29 (0.05,0.53) | 0.22 (0.08,0.35) | 0.08 (-0.03,0.18) | C |

(r) League tables of halos

| A | 1.06 (0.15,7.40) | 4.02 (1.39,11.58) | 4.94 (2.17,11.25) | 8.82 (0.64,121.73) | 8.31 (1.85,37.22) | 10.98 (1.31,91.99) | 19.68 (0.80,483.72) |
| --- | --- | --- | --- | --- | --- | --- | --- |
| 0.95 (0.14,6.63) | H | 3.80 (0.41,34.84) | 4.67 (0.57,38.68) | 8.34 (0.32,219.04) | 7.86 (0.67,91.74) | 10.39 (0.58,185.43) | 18.62 (0.44,789.36) |
| 0.25 (0.09,0.72) | 0.26 (0.03,2.41) | B | 1.23 (0.41,3.73) | 2.20 (0.16,30.35) | 2.07 (0.49,8.74) | 2.73 (0.32,23.66) | 4.90 (0.20,121.83) |
| 0.20 (0.09,0.46) | 0.21 (0.03,1.77) | 0.81 (0.27,2.47) | G | 1.79 (0.13,23.80) | 1.68 (0.38,7.48) | 2.22 (0.29,17.30) | 3.98 (0.17,94.23) |
| 0.11 (0.01,1.56) | 0.12 (0.00,3.15) | 0.46 (0.03,6.29) | 0.56 (0.04,7.47) | F | 0.94 (0.09,9.97) | 1.24 (0.13,11.82) | 2.23 (0.16,31.93) |
| 0.12 (0.03,0.54) | 0.13 (0.01,1.48) | 0.48 (0.11,2.04) | 0.59 (0.13,2.64) | 1.06 (0.10,11.23) | C | 1.32 (0.18,9.53) | 2.37 (0.11,49.85) |
| 0.09 (0.01,0.76) | 0.10 (0.01,1.72) | 0.37 (0.04,3.17) | 0.45 (0.06,3.51) | 0.80 (0.08,7.63) | 0.76 (0.10,5.46) | E | 1.79 (0.13,25.66) |
| 0.05 (0.00,1.25) | 0.05 (0.00,2.28) | 0.20 (0.01,5.07) | 0.25 (0.01,5.94) | 0.45 (0.03,6.41) | 0.42 (0.02,8.89) | 0.56 (0.04,7.98) | D |

(s) League tables of glare

| F | 1.06 (0.27,4.22) | 1.15 (0.25,5.26) | 1.20 (0.47,3.11) | 1.53 (0.56,4.17) | 2.10 (0.58,7.57) | 2.14 (0.57,7.97) | 2.24 (0.51,9.74) |
| --- | --- | --- | --- | --- | --- | --- | --- |
| 0.94 (0.24,3.72) | C | 1.08 (0.24,4.78) | 1.13 (0.28,4.53) | 1.44 (0.30,6.91) | 1.97 (0.57,6.85) | 2.01 (0.56,7.19) | 2.10 (0.50,8.82) |
| 0.87 (0.19,3.97) | 0.92 (0.21,4.09) | B | 1.05 (0.28,3.95) | 1.33 (0.27,6.65) | 1.82 (0.81,4.10) | 1.86 (0.80,4.33) | 1.95 (0.67,5.67) |
| 0.83 (0.32,2.14) | 0.88 (0.22,3.54) | 0.96 (0.25,3.61) | E | 1.27 (0.47,3.45) | 1.74 (0.61,4.98) | 1.78 (0.60,5.29) | 1.86 (0.52,6.65) |
| 0.65 (0.24,1.77) | 0.69 (0.14,3.33) | 0.75 (0.15,3.75) | 0.79 (0.29,2.13) | D | 1.37 (0.34,5.47) | 1.39 (0.34,5.75) | 1.46 (0.31,6.96) |
| 0.48 (0.13,1.72) | 0.51 (0.15,1.77) | 0.55 (0.24,1.24) | 0.57 (0.20,1.65) | 0.73 (0.18,2.93) | G | 1.02 (0.76,1.37) | 1.07 (0.52,2.20) |
| 0.47 (0.13,1.74) | 0.50 (0.14,1.78) | 0.54 (0.23,1.25) | 0.56 (0.19,1.68) | 0.72 (0.17,2.96) | 0.98 (0.73,1.32) | A | 1.05 (0.54,2.02) |
| 0.45 (0.10,1.94) | 0.48 (0.11,1.99) | 0.51 (0.18,1.50) | 0.54 (0.15,1.92) | 0.68 (0.14,3.26) | 0.94 (0.46,1.92) | 0.96 (0.50,1.84) | H |

Note: A: standard monofocal IOLs group; B: bifocal IOLs group; C: AT LISAtri 839MP IOLs group; D: FineVision POD F IOLs group ; E: AcrySof IQ PanOptix IOLs group; F: other new trifocal IOLs group; G: extended depth-of-focus(EDOF) IOLs group; H: enhanced monofocal IOLs group

**Supplementary table 2.** Sorting of SUCRA

1. **UNVA**

| Treatm~t | SUCRA | PrBest | MeanRank |
| --- | --- | --- | --- |
| A | 0.0 | 0.0 | 7.0 |
| B | 21.5 | 0.1 | 5.7 |
| C | 48.1 | 2.2 | 4.1 |
| D | 80.5 | 32.5 | 2.2 |
| E | 74.1 | 10.8 | 2.6 |
| F | 89.1 | 54.1 | 1.7 |
| G | 36.8 | 0.4 | 4.8 |

1. **UIVA**

| Treatm~t | SUCRA | PrBest | MeanRank |
| --- | --- | --- | --- |
| A | 0.7 | 0.0 | 7.9 |
| B | 22.9 | 0.0 | 6.4 |
| C | 58.2 | 8.0 | 3.9 |
| D | 63.3 | 19.5 | 3.6 |
| E | 78.4 | 22.8 | 2.5 |
| F | 68.2 | 18.4 | 3.2 |
| G | 75.8 | 29.4 | 2.7 |
| H | 32.4 | 1.9 | 5.7 |

1. **UDVA**

| Treatm~t | SUCRA | PrBest | MeanRank |
| --- | --- | --- | --- |
| A | 95.1 | 70.3 | 1.3 |
| B | 73.5 | 7.5 | 2.9 |
| C | 48.3 | 1.9 | 4.6 |
| D | 24.2 | 0.3 | 6.3 |
| E | 14.9 | 0.0 | 7.0 |
| F | 7.8 | 0.0 | 7.5 |
| G | 62.0 | 0.4 | 3.7 |
| H | 74.2 | 19.7 | 2.8 |

1. **CNVA**

| Treatm~t | SUCRA | PrBest | MeanRank |
| --- | --- | --- | --- |
| A | 0.0 | 0.0 | 7.0 |
| B | 48.5 | 3.9 | 4.1 |
| C | 51.8 | 14.8 | 3.9 |
| D | 79.4 | 35.9 | 2.2 |
| E | 81.2 | 34.8 | 2.1 |
| F | 66.6 | 10.7 | 3.0 |
| G | 22.5 | 0.0 | 5.6 |

1. **CIVA**

| Treatm~t | SUCRA | PrBest | MeanRank |
| --- | --- | --- | --- |
| A | 0.0 | 0.0 | 8.0 |
| B | 24.4 | 0.0 | 6.3 |
| C | 55.3 | 6.7 | 4.1 |
| D | 73.3 | 13.8 | 2.9 |
| E | 89.9 | 52.9 | 1.7 |
| F | 81.8 | 25.3 | 2.3 |
| G | 52.4 | 1.2 | 4.3 |
| H | 22.9 | 0.1 | 6.4 |

1. **CDVA**

| Treatm~t | SUCRA | PrBest | MeanRank |
| --- | --- | --- | --- |
| A | 80.0 | 7.2 | 2.4 |
| B | 53.3 | 0.0 | 4.3 |
| C | 81.8 | 35.2 | 2.3 |
| D | 24.0 | 0.0 | 6.3 |
| E | 10.2 | 0.0 | 7.3 |
| F | 8.8 | 0.0 | 7.4 |
| G | 49.6 | 0.0 | 4.5 |
| H | 92.3 | 57.6 | 1.5 |

1. **distant Spectacle Independence**

| Treatm~t | SUCRA | PrBest | MeanRank |
| --- | --- | --- | --- |
| A | 23.9 | 0.0 | 5.6 |
| B | 71.8 | 1.4 | 2.7 |
| C | 97.5 | 89.2 | 1.1 |
| D | 23.5 | 0.7 | 5.6 |
| E | 43.8 | 1.0 | 4.4 |
| F | 45.9 | 7.6 | 4.2 |
| G | 43.5 | 0.0 | 4.4 |

1. **intermediate Spectacle Independence**

| Treatm~t | SUCRA | PrBest | MeanRank |
| --- | --- | --- | --- |
| A | 16.4 | 0.0 | 6.0 |
| B | 55.7 | 5.0 | 3.7 |
| C | 80.7 | 53.5 | 2.2 |
| D | 37.3 | 9.0 | 4.8 |
| E | 62.6 | 18.3 | 3.2 |
| F | 37 | 8.6 | 4.8 |
| G | 60.3 | 5.6 | 3.4 |

1. **near Spectacle Independence**

| Treatm~t | SUCRA | PrBest | MeanRank |
| --- | --- | --- | --- |
| A | 2.7 | 0.0 | 6.8 |
| B | 54.7 | 1.9 | 3.7 |
| C | 76.6 | 32.8 | 2.4 |
| D | 51.6 | 13.2 | 3.9 |
| E | 83 | 37.8 | 2.0 |
| F | 52.7 | 14.3 | 3.8 |
| G | 28.7 | 0.0 | 5.3 |

1. **CS under photopic conditions at spatial frequencies of 3cpd**

| Treatm~t | SUCRA | PrBest | MeanRank |
| --- | --- | --- | --- |
| A | 90.5 | 46.2 | 1.7 |
| B | 14.1 | 0.0 | 7.0 |
| C | 17.8 | 0.0 | 6.8 |
| D | 46.8 | 5.3 | 4.7 |
| E | 41.4 | 0.8 | 5.1 |
| F | 47.7 | 3.0 | 4.7 |
| G | 55.9 | 0.4 | 4.1 |
| H | 85.8 | 44.3 | 2.0 |

1. CS under photopic conditions at spatial frequencies of 6cpd

| Treatm~t | SUCRA | PrBest | MeanRank |
| --- | --- | --- | --- |
| A | 81.0 | 30.8 | 2.3 |
| B | 7.5 | 0.0 | 7.5 |
| C | 25.7 | 0.0 | 6.2 |
| D | 72.6 | 27.6 | 2.9 |
| E | 65.2 | 8.8 | 3.4 |
| F | 62.6 | 9.0 | 3.6 |
| G | 15.2 | 0.0 | 6.9 |
| H | 70.3 | 23.8 | 3.1 |

1. CS under photopic conditions at spatial frequencies of 12cpd

| Treatm~t | SUCRA | PrBest | MeanRank |
| --- | --- | --- | --- |
| A | 84.7 | 31.0 | 2.1 |
| B | 11.2 | 0.0 | 7.2 |
| C | 9.4 | 0.0 | 7.3 |
| D | 77.8 | 30.3 | 2.6 |
| E | 39.1 | 0.1 | 5.3 |
| F | 62.8 | 3.4 | 3.6 |
| G | 33.1 | 0.0 | 5.7 |
| H | 81.9 | 35.3 | 2.3 |

1. CS under photopic conditions at spatial frequencies of 18cpd

| Treatm~t | SUCRA | PrBest | MeanRank |
| --- | --- | --- | --- |
| A | 91.0 | 53.0 | 1.6 |
| B | 16.8 | 0.0 | 6.8 |
| C | 6.9 | 0.0 | 7.5 |
| D | 79.7 | 29.9 | 2.4 |
| E | 36.4 | 0.0 | 5.5 |
| F | 55.1 | 0.6 | 4.1 |
| G | 38.5 | 0.0 | 5.3 |
| H | 75.5 | 16.6 | 2.7 |

1. **CS under mesopic conditions at spatial frequencies of 3cpd**

| Treatm~t | SUCRA | PrBest | MeanRank |
| --- | --- | --- | --- |
| A | 86.7 | 14.9 | 1.9 |
| B | 18.7 | 0.0 | 6.7 |
| C | 21.7 | 0.0 | 6.5 |
| D | 54.9 | 2.7 | 4.2 |
| E | 28.7 | 0.0 | 6.0 |
| F | 32.2 | 0.1 | 5.7 |
| G | 60.6 | 0.0 | 3.8 |
| H | 96.6 | 82.2 | 1.2 |

1. **CS under mesopic conditions at spatial frequencies of 6cpd**

| Treatm~t | SUCRA | PrBest | MeanRank |
| --- | --- | --- | --- |
| A | 87.1 | 13.6 | 1.9 |
| B | 24.3 | 0.0 | 6.3 |
| C | 18.4 | 0.0 | 6.7 |
| D | 57.5 | 1.2 | 4.0 |
| E | 24.2 | 0.0 | 6.3 |
| F | 37.9 | 0.0 | 5.3 |
| G | 53.0 | 0.0 | 4.3 |
| H | 97.7 | 85.3 | 1.2 |

1. **CS under mesopic conditions at spatial frequencies of 12cpd**

| Treatm~t | SUCRA | PrBest | MeanRank |
| --- | --- | --- | --- |
| A | 85.5 | 32.4 | 2.0 |
| B | 12.4 | 0.0 | 7.1 |
| C | 4.3 | 0.0 | 7.7 |
| D | 66.5 | 15.5 | 3.3 |
| E | 46.2 | 0.4 | 4.8 |
| F | 66.5 | 8.3 | 3.3 |
| G | 36.0 | 0.0 | 5.5 |
| H | 82.6 | 43.2 | 2.2 |

1. **CS under mesopic conditions at spatial frequencies of 18cpd**

| Treatm~t | SUCRA | PrBest | MeanRank |
| --- | --- | --- | --- |
| A | 67.5 | 10.7 | 2.9 |
| B | 16.3 | 0.0 | 6.0 |
| C | 1.6 | 0.0 | 6.9 |
| D | 96.4 | 83.2 | 1.2 |
| E | 52.2 | 0.1 | 3.9 |
| F | 77.6 | 5.9 | 2.3 |
| G | 38.5 | 0.0 | 4.7 |

1. **Halos**

| Treatm~t | SUCRA | PrBest | MeanRank |
| --- | --- | --- | --- |
| A | 91.6 | 47.4 | 1.6 |
| B | 56.9 | 0.1 | 4.0 |
| C | 32.6 | 0.1 | 5.7 |
| D | 18.9 | 1.9 | 6.7 |
| E | 28 | 0.7 | 6.0 |
| F | 36.4 | 2.9 | 5.5 |
| G | 49.6 | 0.0 | 4.5 |
| H | 86 | 46.9 | 2 |

1. **glare**

| Treatm~t | SUCRA | PrBest | MeanRank |
| --- | --- | --- | --- |
| A | 26.5 | 0.1 | 6.1 |
| B | 68.6 | 24.6 | 3.2 |
| C | 68.9 | 28.3 | 3.2 |
| D | 46.2 | 7.2 | 4.8 |
| E | 63.4 | 10.6 | 3.6 |
| F | 73.1 | 27.6 | 2.9 |
| G | 28.2 | 0.1 | 6 |
| H | 25.2 | 1.4 | 6.2 |
